# Identification of a Novel *Staphylococcus aureus* Two-Component Leukotoxin Using Cell Surface Proteomics

**DOI:** 10.1371/journal.pone.0011634

**Published:** 2010-07-16

**Authors:** Christy L. Ventura, Natalia Malachowa, Carl H. Hammer, Glenn A. Nardone, Mary Ann Robinson, Scott D. Kobayashi, Frank R. DeLeo

**Affiliations:** 1 Laboratory of Human Bacterial Pathogenesis, Rocky Mountain Laboratories, National Institute of Allergy and Infectious Diseases, National Institutes of Health, Hamilton, Montana, United States of America; 2 Research Technologies Branch, National Institute of Allergy and Infectious Diseases, National Institutes of Health, Rockville, Maryland, United States of America; University of Liverpool, United Kingdom

## Abstract

*Staphylococcus aureus* is a prominent human pathogen and leading cause of bacterial infection in hospitals and the community. Community-associated methicillin-resistant *S. aureus* (CA-MRSA) strains such as USA300 are highly virulent and, unlike hospital strains, often cause disease in otherwise healthy individuals. The enhanced virulence of CA-MRSA is based in part on increased ability to produce high levels of secreted molecules that facilitate evasion of the innate immune response. Although progress has been made, the factors that contribute to CA-MRSA virulence are incompletely defined. We analyzed the cell surface proteome (surfome) of USA300 strain LAC to better understand extracellular factors that contribute to the enhanced virulence phenotype. A total of 113 identified proteins were associated with the surface of USA300 during the late-exponential phase of growth *in vitro*. Protein A was the most abundant surface molecule of USA300, as indicated by combined Mascot score following analysis of peptides by tandem mass spectrometry. Unexpectedly, we identified a previously uncharacterized two-component leukotoxin–herein named LukS-H and LukF-G (LukGH)-as two of the most abundant surface-associated proteins of USA300. Rabbit antibody specific for LukG indicated it was also freely secreted by USA300 into culture media. We used wild-type and isogenic *lukGH* deletion strains of USA300 in combination with human PMN pore formation and lysis assays to identify this molecule as a leukotoxin. Moreover, LukGH synergized with PVL to enhance lysis of human PMNs *in vitro*, and contributed to lysis of PMNs after phagocytosis. We conclude LukGH is a novel two-component leukotoxin with cytolytic activity toward neutrophils, and thus potentially contributes to *S. aureus* virulence.

## Introduction

*Staphylococcus aureus* is a leading cause of human bacterial infections worldwide. The organism can cause a wide range of diseases, including superficial skin and soft tissue infections, as well as invasive diseases such as pneumonia, bacteremia, endocarditis, and joint infections (reviewed in [1]). The high prevalence of infections is confounded by the ability of the pathogen to readily acquire genetic elements that confer resistance to antibiotics. Although methicillin-resistant *S. aureus* (MRSA) remains a significant problem for healthcare facilities in most industrialized countries, an MRSA strain known as USA300 is the most abundant cause of bacterial infections outside of healthcare facilities in the United States [2]–[4]. The ability of USA300 to cause infections in otherwise healthy individuals suggests the strain has enhanced capacity to circumvent killing by the innate immune system—a notion confirmed by studies with human neutrophils [5]. The ability of CA-MRSA strains such as USA300 to produce relatively high levels of secreted molecules such as phenol-soluble modulins (PSMs) provides an explanation in part for the enhanced ability of these pathogens to avoid destruction by neutrophils [6], [7].

Despite this recent progress, our understanding of CA-MRSA virulence mechanisms is incomplete, largely because *S. aureus* produces many molecules that can potentially contribute to immune evasion and virulence [8]. To gain a comprehensive view of the molecules that potentially promote USA300 virulence, we evaluated the surface proteome of this pathogen. Unexpectedly, we identified an uncharacterized two-component leukotoxin—herein named LukS-H and LukF-G—bound to the surface of USA300, and used USA300 wild-type and isogenic *lukGH* mutant strains to verify its ability to function as a leukotoxin.

## Results and Discussion

### Isolation of peptides from USA300 surface proteins

We optimized the approach described by Rodriguez-Ortega et al. to determine the cell surface proteome (surfome) of USA300/LAC [9]. Because *S. aureus* cell wall-associated proteins are expressed maximally during exponential growth [10], we used cultures grown to late-exponential phase of growth. USA300 surface proteins were removed by digestion with trypsin in the presence of sucrose and DTT, which make surface proteins more accessible to trypsin [9]. One concern with this approach is that proteolytic digestion of the bacterial surface proteins has the potential to cause cell lysis, either through destabilization of the membrane as a result of loss of membrane proteins or through activation of bacterial autolytic and/or proteolytic proteins. To determine if trypsin decreased viability of LAC, we plated the bacteria before and after digestion with trypsin (F. S1A). There was a slight increase in CFU/ml after exposure to trypsin; this phenomenon was likely due to decreased clumping of bacteria following removal of the proteinaceous surface clumping factors (Fig. S1A). Although exposure of bacteria to trypsin for an extended period of time (21 h) resulted in lysis of bacteria (data not shown), there was no decrease in bacterial viability following the relatively short trypsin incubation period used here to remove surface proteins (Fig. S1A). Separation of a representative 1-h tryptic digest by Tricine SDS-PAGE demonstrated that the surface proteins were digested into small peptides (Fig. S1B).

### Identification of USA300 surface proteins

**Figure 1:**
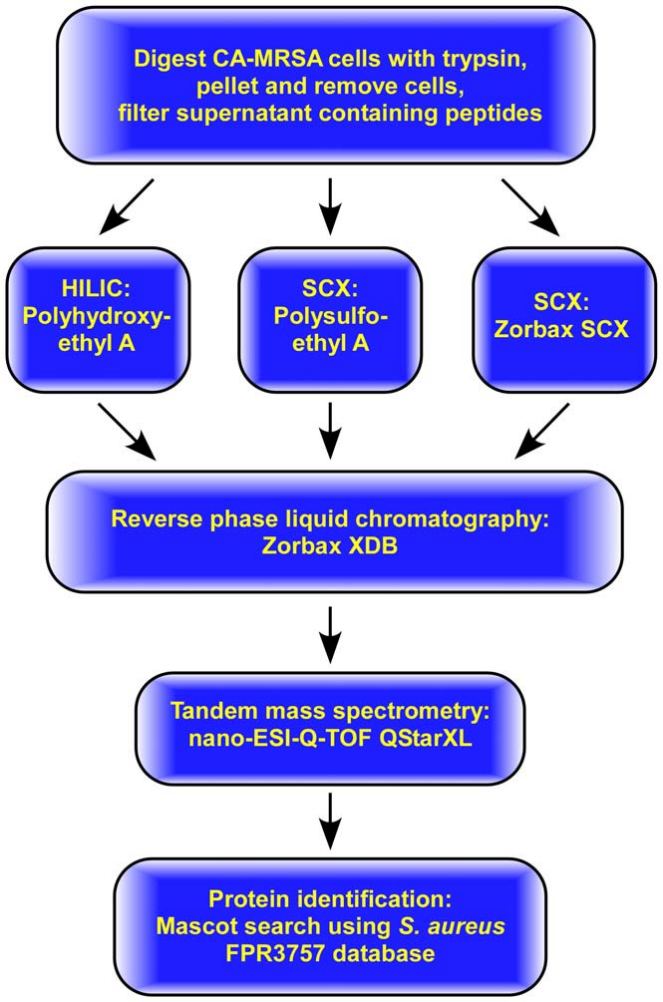
Workflow diagram for surface proteomics.

**Figure 2:**
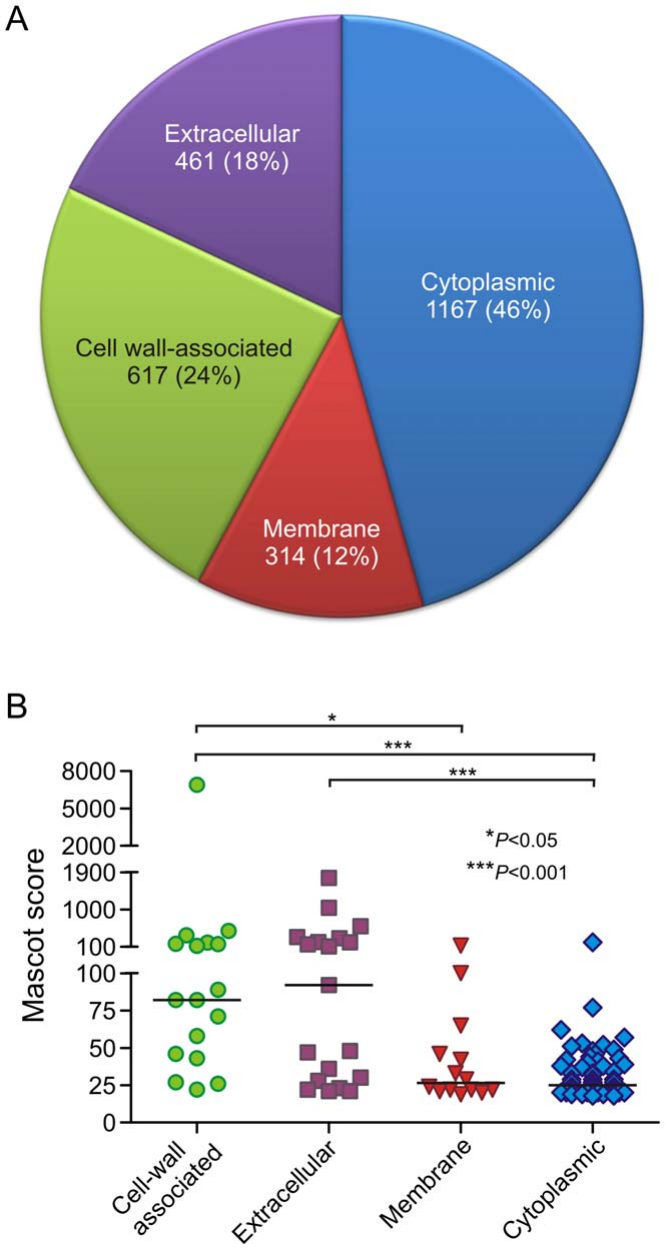
Relative abundance of proteins identified on the surface of USA300. PSORT v2.0 was used to assign identified proteins to subcellular compartments. (A) Total number of peptides assigned to proteins in each cellular compartment. A total of 2559 peptides were matched to proteins in the RSUE database. (B) Each data point in the scatter plot represents one protein. The line for each cellular location represents the median Mascot score for that compartment. Mascot scores were evaluated by a Kruskal-Wallis test with Dunn's post-test to compare the relative confidence of protein identifications between subcellular compartments.

Peptides recovered from the surface of USA300 were subjected to fractionation and tandem mass spectrometry (LC-MS/MS) as outlined in Fig. 1. Using this approach, we identified 113 proteins associated with the surface of USA300 during late–exponential phase of growth (Fig. 2, and Tables S1 and S2). Of these proteins, 17 are predicted to be associated with the cell wall using a combination of PSORT [11] and previous studies (Table S1). In addition to the 17 cell wall-associated proteins, we recovered 19 extracellular and 14 membrane proteins from the surface protein preparations of USA300. Three of the 19 putative extracellular proteins (LukS-H, IsaA, and LukF-G) were also found in high abundance on the cell surface during exponential or stationary phase of growth ([12]; this study). As shown in Fig. 2A, 54% of the peptides (“queries” in Table S2) identified by our MS/MS analysis could be assigned to only 50 proteins (17 cell wall-associated, 19 extracellular, and 14 membrane); the number of peptides assigned to a protein is a direct measure of the abundance of that protein in the sample. In contrast, Mascot assigned the remaining 46% of the identified peptides to 63 proteins that normally reside in the cell cytoplasm (Table S1 and Fig. 2A). As such, the confidence with which these proteins were identified was significantly lower than that for cell wall-associated and/or extracellular proteins (Fig. 2B).

### Cell wall-associated proteins

**Figure 3:**
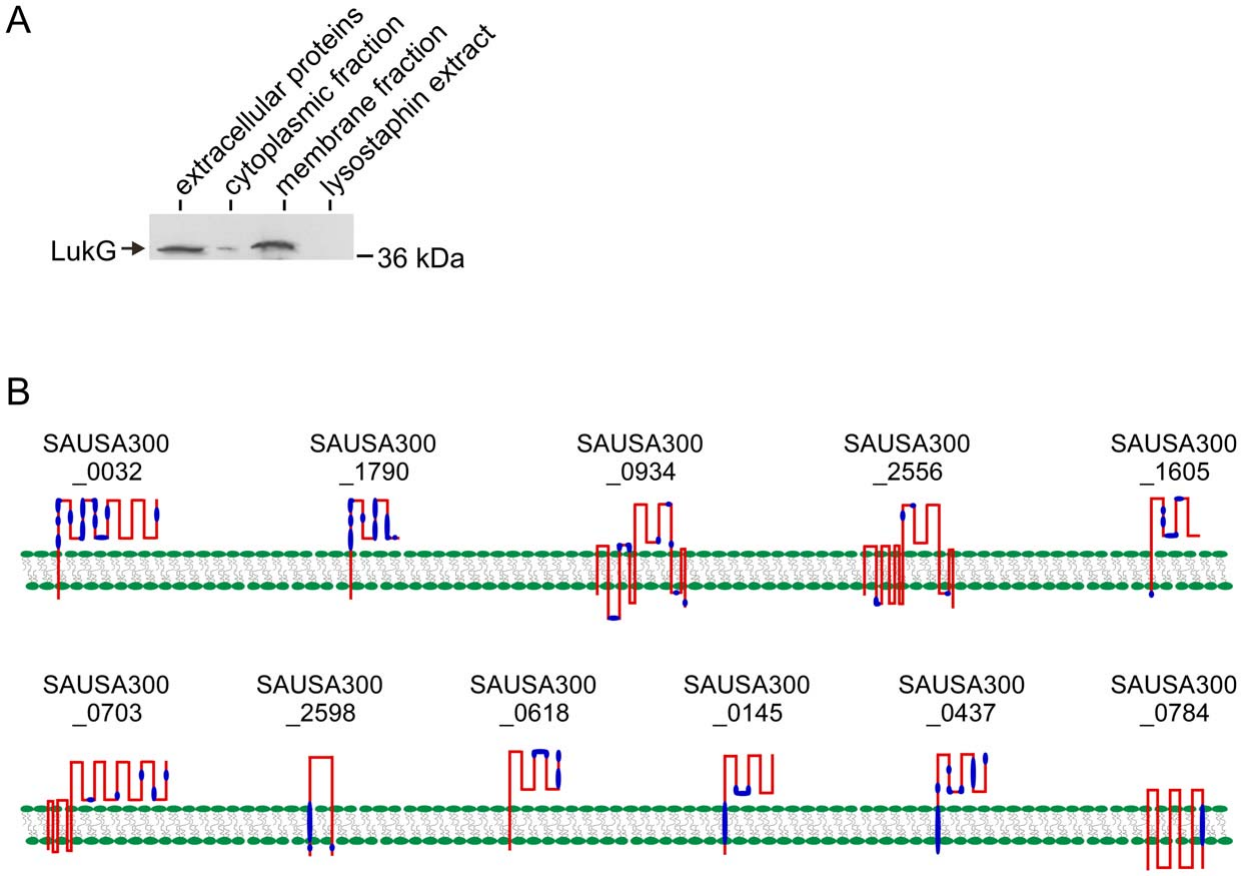
Membrane association of LukG and predicted topology for putative membrane proteins. (A) Immunoblot analysis of LukG secreted into the culture media (extracellular) or associated with subcellular fractions of USA300. Subcellular fractions were prepared as described in Materials and Methods. (B) Predicted membrane proteins identified by surface proteomics. Blue regions indicate areas of peptide coverage.

The classical Gram-positive peptide, LPxTG, which covalently anchors proteins in the cell wall peptidoglycan, is present in 12 of the 17 cell wall-associated proteins identified by our analysis (Table S1). The LysM domain, which non-covalently links proteins to the cell surface, is present in 2 of these 17 proteins, as well as one membrane protein (EbpS). The most abundant protein on the surface of USA300 was immunoglobulin G binding protein A (protein A or Spa), based upon the number of unique peptides identified (46), the number of total peptides matched (273), and the combined Mascot score (6910) (Tables S1 and S2). This finding is in agreement with previously published studies of *S. aureus* surface proteins [13]–[16]. Interestingly, two previously unidentified/uncharacterized proteins encoded by open reading frames annotated in the FPR3757 genome as *SAUSA300_1975* and *SAUSA300_1974* were the 2^nd^ and 7^th^ most abundant proteins on the surface of USA300 (Table S1). *SAUSA300_1974* and *SAUSA300_1975* are predicted to encode LukF and LukS subunits of a two-component leukotoxin, which is present and highly conserved among all sequenced *S. aureus* strains (Fig. S2A and S2B). Here we designate *SAUSA300_1974* as *lukF-G* (or *lukG*) and *SAUSA300_1975* as *lukS-H* (or *lukH*), and the two-gene operon as *lukGH*. Neither LukF-G nor LukS-H (hereafter called LukG and LukH, respectively) contains LPxTG or LysM cell wall anchoring domains, though both were highly abundant on the surface of *S. aureus* during late-exponential growth *in vitro* (Table S1). We verified by immunoblot analysis of subcellular fractions of USA300 that LukG is associated with the detergent-soluble membrane fraction (Fig. 3A).

The majority of the cell wall-associated proteins identified by our analysis have been described previously (reviewed in [17]). Interestingly, iron-regulated surface determinants A (IsdA) and B (IsdB) were the 6^th^ and 21^st^ most abundant proteins on the cell surface, as predicted by Mascot score (Table S1). Expression of the Isd proteins has been shown to occur only in iron-limited conditions [18], unlike the rich TSB medium in which USA300 was cultured for this study.

### Extracellular/secreted proteins

The methodology we employed to remove proteins from the surface of USA300 was not specific to conventionally defined surface proteins (i.e., cell wall-associated and membrane proteins). Thus, we identified proteins in the process of being secreted into the culture medium as well. Five of the 10 most abundant proteins are putative or proven secreted proteins, and 3 others are associated with both the cell wall and the extracellular milieu. LukG and LukH are among the most abundant extracellular proteins identified by our analysis (in this category ranked 1^st^ and 3^rd^ in abundance by Mascot score) (Table S1). Gamma-hemolysin subunit A (HlgA) was the only other two-component leukotoxin subunit identified as associated with the cell surface (Table S1). Expression of delta-hemolysin and other secreted proteins, many of which were identified here, is largely dependent on upregulation of the global gene regulator *agr*, which occurs *in vitro* at the transition from exponential to stationary phase of growth [10]. Thus, our finding that delta-hemolysin is associated with the surface of *S. aureus* suggests bacteria were beginning to make the transition from exponential growth (high production of cell wall-associated proteins) to stationary phase of growth (production of secreted proteins).

### Membrane proteins

We identified 14 membrane proteins on the surface of *S. aureus* in our surfome analysis. Membrane topology analysis using HMMTOP showed that many of the peptides assigned to membrane proteins were located on the exterior of the cell membrane (Fig. 3B). Six of the membrane proteins are putative components of as yet uncharacterized ABC transport systems, and one is predicted to be an amino acid efflux protein. In addition, we found MecA (SAUSA300_0032) on the surface of USA300 strain LAC (Fig. 3B). MecA is the penicillin-binding protein that confers resistance to β-lactam antibiotics in MRSA strains. We also identified CapA (SAUSA300_2598), which is encoded by the first gene in the staphylococcal capsule operon (Table S1). *S. aureus* capsule operon expression is tightly regulated by *agr* and is usually not present during the exponential phase of growth [19]. These findings indicate that the proteomic approach utilized here was sufficient to identify surface-exposed peptides from membrane proteins.

### Cytoplasmic proteins

Based on the number of proteins identified by surfome analysis, the majority are putative or proven cytoplasmic proteins (Table S1). However, as mentioned above, these proteins were significantly less abundant on the surface than were those predicted to be cell wall-associated and/or extracellular (Fig. 2B and Tables S1 and S2). Ribosomal proteins comprise 25 of the 63 (40%) cytoplasmic proteins identified by our analysis, presumably as a result of normal cell turnover. Although there was no decrease in CFU/ml following digestion of surface proteins with trypsin, it is likely that some cell lysis occurred during digestion or subsequent processing, perhaps accounting in part for presence of cytoplasmic proteins in our cell surface proteome analysis.

### LukGH is a novel *S. aureus* leukotoxin

Using the combined Mascot score as a measure of relative abundance, the 2^nd^ and 7^th^ most abundant USA300 proteins identified by surface proteomics were LukH and LukG, respectively (Table S1). As noted above, *lukG* and *lukH* are present in all sequenced *S. aureus* strains and there is limited allelic variation among the strains (97–100% nucleotide identity) (Fig. S2A). USA300 strains contain up to 4 operons encoding known or predicted two-component leukotoxins [20]. LukGH shares significant homology with other staphylococcal leukotoxins, including PVL and HlgABC (see figure 1 in ref. [21]) (Fig. S2C). LukG is 37% identical to HlgB and 36% identical to LukF-PV, and LukH is 26–28% identical to LukS-PV, HlgA and HlgC, based on a CLUSTALW2 analysis of inferred amino acids from strain FPR3757 (Fig. S2C). However, LukGH has not been previously characterized and its function remains to be determined. Based on homology and predicted function as a secreted toxin, LukGH should be secreted into the extracellular milieu during post-exponential growth of *S. aureus in vitro* [10]. However, Luong et al. describe expression of *lukG* (N315–1812) and *lukH* (N315–1813) during late-exponential phase of growth [22]. Further, we demonstrated previously that *lukH* was highly expressed during PMN phagocytosis (labeled as aerolysin/leukocidin family protein or *lukM* in those reports), and following exposure to neutrophil azurophilic granule proteins [5], [23], [24].

**Figure 4:**
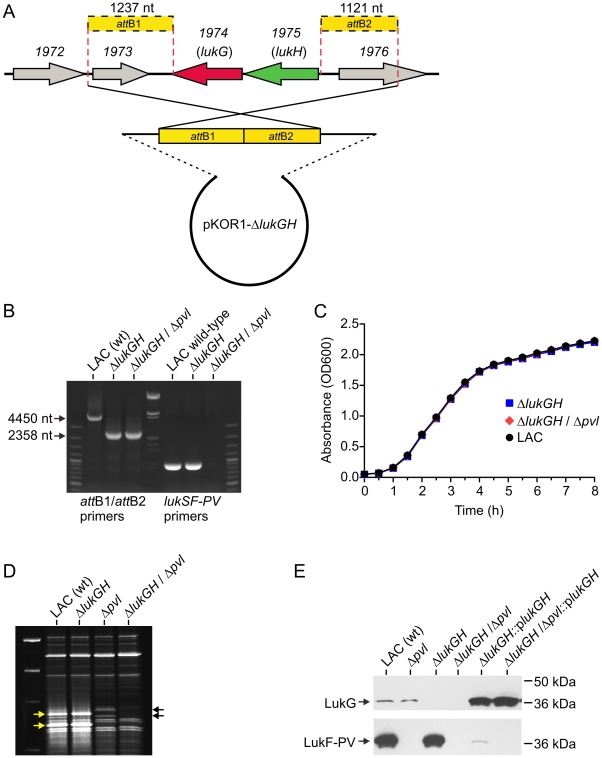
Construction and characterization of isogenic USA300 *lukGH* deletion (Δ*lukGH*) strains. (A) Schematic of deletion strategy for generation of Δ*lukGH* and (B) confirmation of isogenic Δ*lukGH* and Δ*lukGH*/Δ*pvl* strains by PCR as described in Methods. (C) Growth of LAC wild-type, Δ*lukGH* and Δ*lukGH*/Δ*pvl* strains. (D) Exoprotein profile of LAC culture supernatants from early-stationary phase of growth (6 h). Proteins were resolved by SDS-PAGE and stained with Sypro Ruby. Black arrows correspond to LukG and LukH and yellow arrows denote LukF-PV and LukS-PV. (E) Immunoblot analysis of LukG and LukF-PV from culture supernatants of LAC wild-type-, isogenic gene deletion-, and complemented gene deletion strains as indicated.

As a first step toward determining the function of LukGH, we constructed isogenic *lukGH* (Δ*lukGH*) and *lukGH*/*lukSF-PV* (Δ*lukGH/*Δ*pvl*) deletion mutants in USA300 (LAC) using the counterselectable marker system developed by Bae and Schneewind (Fig. 4A) [25]. We generated the USA300Δ*lukGH/*Δ*pvl* strain in the genetic background of a previously described USA300Δ*pvl* strain [23]. PCR analysis of genomic DNA isolated from the wild-type and mutant strains confirmed *lukGH* were deleted from Δ*lukGH* and Δ*lukGH/*Δ*pvl*, and all strains exhibited virtually identical growth in TSB media (Fig. 4B and C). Since LukGH has identity to other two-component leukotoxins that are normally secreted into culture media, we assessed exoprotein profiles of each strain cultured to early-stationary phase of growth in CCY medium, which promotes high production of PVL (Fig. 4D). Protein bands corresponding to LukF-PV and LukS-PV (yellow arrows) were clearly present in the media in which LAC wild-type and Δ*lukGH* strains were cultivated, and absent in the media from the Δ*pvl* and Δ*lukGH/*Δ*pvl* cultures (Fig. 4D). Similarly, bands corresponding to LukG and LukH, as indicated by black arrows, were present in the LAC wild-type and Δ*pvl* supernatants, and absent from supernatants derived from Δ*lukGH* and Δ*lukGH/*Δ*pvl* cultures (Fig. 4D). Rabbit IgG specific for LukG identified the protein in culture supernatants, indicating the leukotoxin is at least in part secreted (Fig. 4E). Δ*lukGH* and Δ*lukGH/*Δ*pvl* were complemented with *lukGH* encoded on a plasmid (Δ*lukGH*::p*lukGH* and Δ*lukGH/*Δ*pvl*::p*lukGH*, respectively), thereby restoring production of the leukotoxin (as assessed by immunoblot for LukG) (Fig. 4E). All strains produced equivalent amounts of alpha-hemolysin or protein A, and had comparable levels of beta-hemolysis on blood agar (data not shown), suggesting that the global regulator *agr* remained intact during strain passage and mutagenesis.

### LukGH has cytolytic activity toward human PMNs

**Figure 5:**
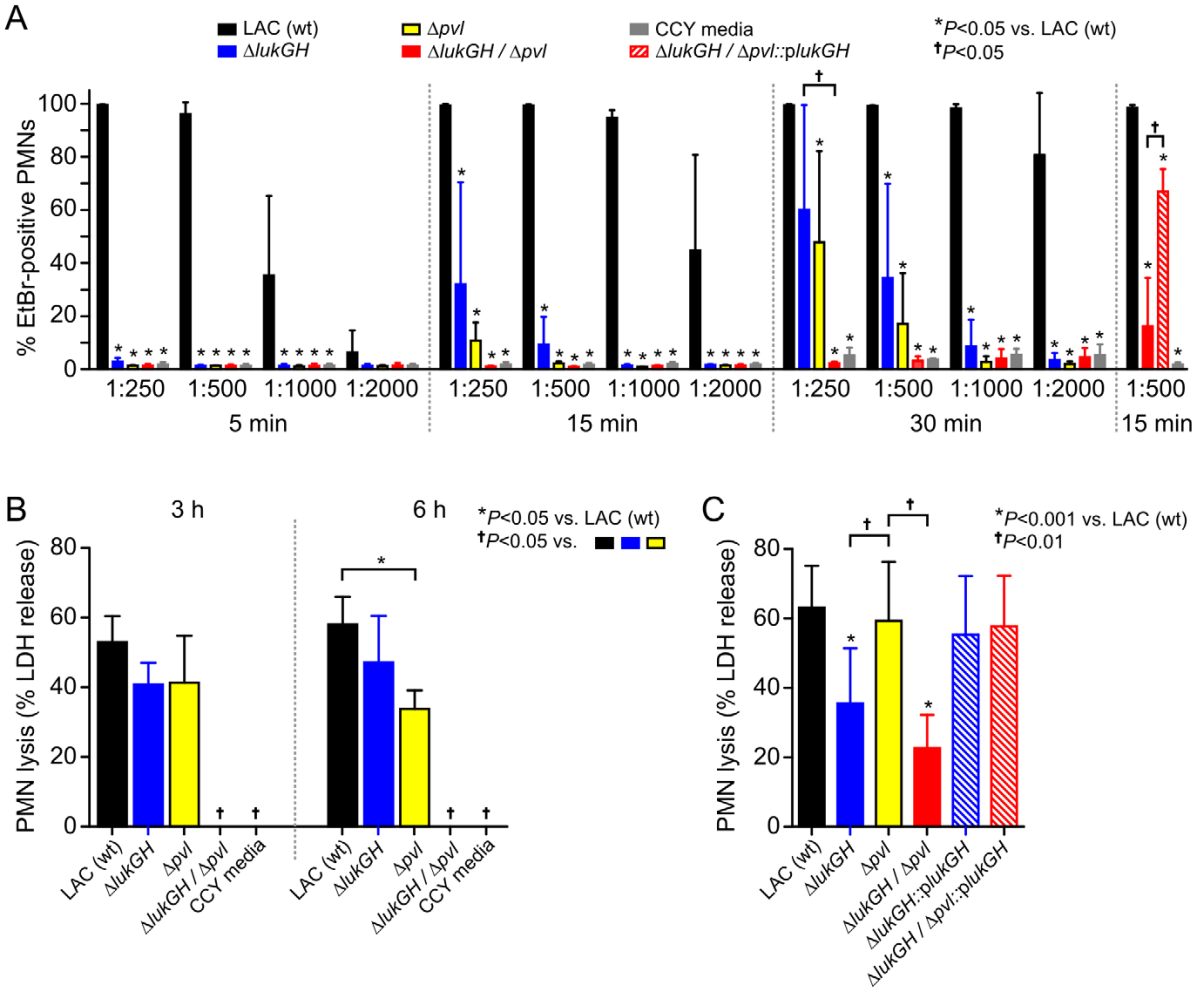
LukGH contributes to PMN plasma membrane pore formation and lysis. (A) Permeability of human PMNs exposed to culture supernatants from LAC wild-type-, isogenic gene deletion-, or complemented gene deletion strains. (B) Capacity of LAC wild-type and mutant CCY culture supernatants to cause PMN lysis. Dilution of culture supernatants was 1∶250. (C) PMNs were cultured with LAC strains for 6 h and PMN lysis was determined by release of LDH. Statistical analyses for panels (A, B, and C) were performed using repeated-measures ANOVA or one-way ANOVA and Tukey's posttest. *P<0.05 versus LAC or †*P*<0.05 as indicated. Results are the mean ± standard deviation of 4 (panel A), 3 (panel B) or 4–10 (panel C) PMN donors.

Because we detected LukGH in the culture media, we compared the ability of CCY culture supernatants from LAC wild-type, Δ*pvl*, Δ*lukGH*, and Δ*lukGH/*Δ*pvl* strains to cause formation of plasma membrane pores in human PMNs (Fig. 5A). Culture supernatants from Δ*pvl*, Δ*lukGH* or Δ*lukGH/*Δ*pvl* strains had significantly decreased capacity to cause formation of membrane pores compared with those from the wild-type strain (Fig. 5A). Using conditions in which pore-forming capacity is retained in either of the single operon deletion strains (30 min incubation using more concentrated supernatants), culture supernatants from the Δ*lukGH/*Δ*pvl* strain typically caused little or no formation of membrane pores (Fig. 5A). Complementation of the Δ*lukGH/*Δ*pvl* strain with a plasmid containing *lukGH* restored a significant level of pore-forming capacity, thereby demonstrating that LukGH contributes to neutrophil plasma membrane pore-formation (Fig. 5A, far right panel).

Although we observed significantly reduced pore formation in PMNs exposed to the highest concentration of culture supernatants from Δ*pvl* or Δ*lukGH* strains (1∶250 dilution at 5 or 15 min) (Fig. 5A), there was only a limited corresponding decrease in PMN lysis (as measured by LDH release) (Fig. 5B). For example, supernatants from cultures of Δ*pvl* or Δ*lukGH* strains retained most of their cytolytic capacity after 3 h of incubation with human PMNs—although there was a trend for decreased LDH release (Fig. 5B). This finding is consistent with our studies indicating that PMN pore formation does not necessarily correlate with cytolysis [26]. By contrast, culture supernatants from the Δ*lukGH/*Δ*pvl* strain had zero cytolytic capacity, indicating synergy or cooperativity between PVL and LukGH (Fig. 5B). We note that culture supernatants from the Δ*pvl* strain had significantly reduced cytolytic capacity by 6 h (LDH release was 58.0±7.9% for wild-type supernatants and 33.8±5.4% for those from the Δ*pvl* strain, *P*<0.05), but the differential supernatant lysis between the two mutant strains is likely due to the selective overproduction of PVL in CCY media (Fig. 4D) [26], [27]. The finding that there is synergy between PVL and LukGH merits further investigation.

Inasmuch as we identified LukGH by surface proteome analysis, we tested the hypothesis that surface-associated LukGH contributes to rapid lysis of human PMNs after phagocytosis of USA300 [5]. Lysis of PMNs 6 h after phagocytosis of the Δ*pvl* strain was comparable to that caused by the wild-type USA300 strain (LDH release was 63.2±12.0% and 59.3±17.0% for the wild-type and Δ*pvl* strains, n = 10) (Fig. 5C). These findings are consistent with our previous studies demonstrating PVL does not contribute to lysis of PMNs after phagocytosis [5], [23]. By comparison, Δ*lukGH* or Δ*lukGH/*Δ*pvl* strains had significantly reduced capacity to cause lysis after uptake by PMNs (e.g., LDH release was 35.5±15.9% following phagocytosis of Δ*lukGH*, *P*<0.01 versus LAC wild-type) (Fig. 5C). The complemented mutant strains, Δ*lukGH*::p*lukGH* and Δ*lukGH/*Δ*pvl*::p*lukGH*, caused PMN lysis at a level similar to that of the wild-type strain (Fig. 5C). Although it was not possible to differentiate the relative contribution of surface-associated versus freely secreted LukGH in this process, there is a relative high abundance of surface-associated LukGH, whereas the other two-component leukotoxins are either not present on the cell surface (LukS-PV, LukF-PV, LukD, LukE, HlgB, HlgC) or are present in much lower abundance (i.e., HlgA) (Table S1). Taken together, these data demonstrate that LukGH has potent cytolytic activity toward human neutrophils.

### Concluding remarks

Rodriguez-Ortega et al. used a surface proteomics approach to identify proteins present on the surface of group A *Streptococcus*, some of which are potential vaccine candidates [9]. More recently, Solis et al. evaluated the surface proteome of *S. aureus* strain COL using methodology that reduces contamination with proteins from the cytoplasm [28]. Indeed, we recovered a higher percentage of peptides corresponding to cytoplasmic proteins than did Solis et al., but Mascot scores for the corresponding cytoplasmic proteins were significantly lower than those of cell-wall associated, extracellular, or membrane proteins (Fig. 2B). Many of the proteins identified here by our surface proteome analysis, such as Atl, ClfB, MecA, IsaA, IsdH, SasF, and Spa, were also reported to be surface-associated by Solis et al. [28]. However, to our knowledge, LukG and LukH have not been identified previously as exoproteins.

*S. aureus* has the capacity to produce several homologous two-component leukotoxins, including gamma-hemolysin (Hlg, encoded by *hlgA, hlgB, and hlgC*) [29], [30], leukotoxin D and E (LukDE, encoded by *lukD* and *lukE*) [31], LukM and LukF'-PV [32], and PVL (encoded by *lukS-PV* and *lukF-PV* within specific bacteriophage) [33]–[39]. These toxins assemble as pore-forming multimers on the surface of susceptible target cells, such as neutrophils, monocytes, and macrophages, and can thereby alter host cell function or cause cytolysis [40]–[43]. Genes encoding Hlg and LukDE are present in the vast majority of sequenced *S. aureus* strains or clinical isolates tested [44], whereas those encoding PVL exist in 5% or less of all clinical isolates [44]–[46]. *lukM* and *lukF*'*-PV* are associated with *S. aureus* strains that cause mastitis in cows, ewes, and goats, but have not been found in human clinical isolates [44]. In addition, all sequenced *S. aureus* strains contain open reading frames (ORFs) encoding a potential two-component leukocidin that has remained uncharacterized. The *lukF* subunit of this potential leukotoxin (ORF *SAUSA300_1974* in USA300 strain FPR3757) is annotated as a leukocidin/hemolysin toxin family protein with 38–39% identity to LukD, HlgB, HlgC, and LukF-PV. The *lukS* subunit (*SAUSA300_1975* in FPR3757) is annotated as an aerolysin/leukocidin family protein with 35% identity to LukM and 31% identity to LukS-PV. We have previously shown that this gene is up-regulated in strain MW2 (USA400) during phagocytic interaction with human neutrophils (called *lukM* in that study) [23].

Herein we used a surface proteomics approach to identify this previously uncharacterized leukotoxin (LukGH) as an abundant exoprotein of USA300. LukGH was both surface-associated and present in USA300 culture media. Using isogenic USA300 mutants, we discovered that this novel leukotoxin has potent cytolytic activity toward neutrophils and can work synergistically with PVL to cause PMN lysis. Given the high abundance of LukGH in our surface protein preparations, and the critical role that neutrophils play in host defense against *S. aureus* infection, it is tempting to speculate that the toxin contributes to the enhanced virulence phenotype of USA300. Studies in animal infection models to demonstrate a role for LukGH in *S. aureus* virulence are ongoing.

## Materials and Methods

### Ethics statement

Human neutrophils were isolated from heparinized venous blood of healthy donors in accordance with a protocol approved by the NIAID Institutional Review Board for human subjects. Donors were informed of the procedure risks and provided written consent prior to enrollment.

### Bacterial strains and culture conditions

We used USA300 strain LAC (Los Angeles County clone) for surfome analysis. LAC has been described and characterized previously [5] and is representative of the USA300 epidemic clone [47]. LAC was grown in trypticase soy broth (TSB) or CCY media (3% yeast extract, 2% Bacto-casamino acids, 0.21 M sodium pyruvate, 44 mM dibasic sodium phosphate, 3 mM monobasic potassium phosphate, pH 6.7) in a flask-to-media volume ratio of 5∶1 at 225 rpm at 37°C. LAC was cultured overnight in TSB, diluted 1/200 into fresh media, and subcultured in either TSB or CCY using the same conditions until the desired growth phase was obtained (late-exponential phase in TSB for proteomics, early-stationary phase in CCY for culture supernatant assays with PMNs, and mid-exponential phase in TSB for PMN lysis assays with intact bacteria). Growth curve analysis in TSB was performed in triplicate for each strain, and bacteria were plated for enumeration on trypticase soy agar plates.

### Sample preparation for proteomics analysis

USA300/LAC was cultured in TSB to late-exponential phase of growth (OD_600_ = 0.85−1.0) in 25- or 100-ml volumes. Bacteria were harvested by centrifugation at 4,000 *g* for 15 min at 4°C, and washed 3 times with a volume of chilled 50 mM Tris, pH 7.5, that was equivalent to the original culture volume. The pellet was resuspended in 1 ml (25 ml culture) or 3 ml (100 ml culture) of chilled 50 mM Tris, pH 7.5, and aliquoted (1 ml/tube) into 1.5-ml Protein Lo-Bind tubes (Eppendorf North America, Westbury, NY). Bacteria were pelleted at 16,100 *g* for 5 min at 4°C and resuspended in 0.3 ml digestion buffer (0.6 M sucrose buffered with 50 mM Tris, pH 7.5,) containing 2 mM dithiothreitol (DTT), and 10–12 µg mass spectrometry-grade trypsin (Promega, Madison, WI). Sucrose was included in the digestion buffer to facilitate swelling of the bacterial cells, which enhances trypsin digestion of surface proteins [9]. Bacteria were digested with trypsin for 1 h at 37°C. A 25× solution of SIGMAFast protease inhibitor cocktail (Sigma-Aldrich Company, St. Louis, MO) was prepared in digestion buffer; the cocktail was added to a final concentration of 1× following trypsin digestion. An aliquot of the digested sample before and after incubation at 37°C was diluted serially in 50 mM Tris, pH 7.5, and plated on trypticase soy agar plates to assess bacterial viability. Following trypsin digestion, bacteria were pelleted by centrifugation at 8,000 *g* for 10 min at 4°C. The supernatant containing tryptic peptides from USA300 surface proteins was clarified/sterilized by using a Microcon YM-100 centrifugal filter unit (Millipore, Billerica, MA) at 4°C for 25 min. The filtrate was frozen at −80°C until further processing.

### Fractionation of tryptic peptides

Several approaches were used to prepare enriched peptide fractions. Trifluoroacetic acid (TFA) was added to the tryptic peptides from three 25-ml cultures to a final concentration of 0.2% and extracted using a reverse phase polymer peptide trap cartridge (Michrom Bioresources, Auburn, CA) to remove interfering salts and sucrose. The dried sample was dissolved in 50 µl of 50 mM formic acid/75% isopropanol and applied to polyhydroxyethyl A 10-µl solid phase extraction tip (Glygen Corporation, Columbia, MD). The analytes were eluted with 10 µl of 15 mM ammonium acetate, pH 3.5/3% acetonitrile, 0.1% TFA and fractionated by reverse phase chromatography using a Zorbax XDB 5 µm C18, 0.5 mm ×150 mm column on an 1100 series capillary HPLC system (Agilent Technologies, Santa Clara, CA). Linear gradients were run from 0.1% TFA/3% acetonitrile to 60% acetonitrile. Column effluent was monitored at 220 and 280 nm. The 12-µl column fractions were dried and dissolved in 4.5 µl of 0.1% acetic acid/50% methanol for analysis by mass spectrometry. In other experiments, the tryptic peptides from five 25-ml cultures were adjusted to pH 2.8 by the addition of 10% formic acid and applied to a Polysulfoethyl A 10-µl solid phase extraction tip (Glygen Corporation). Following a 100-µl wash with 0.2% formic acid/5% acetonitrile, the resin was eluted with 20 µl of 0.01, 0.05, 0.1, 0.5 and 1 M NaCl in the same solvent. The batches were dried, dissolved in 10 µl of 0.1% TFA/3% acetonitrile and each submitted to reverse phase chromatography as described. Ion exchange chromatography was also performed with a Zorbax SCX, 5 µm 2.1 mm ×50 mm column on a 1100 series analytical HPLC system (Agilent Technologies) using the peptides from eleven 25-ml cultures. The column was developed with a gradient of NaCl from 0.01 to 1 M in 0.2% formic acid/5% acetonitrile. The 0.2-ml fractions were combined into seven separate pools which were qualitatively based on the UV peak patterns at 220 nm. After drying under vacuum 5 of the 7 pools were dissolved in 10 µl of 0.1% TFA/3% acetonitrile and submitted to reverse phase chromatography. The two higher salt pools were dissolved in 200 µl 0.1% TFA and desalted by reverse phase cartridge as described prior to capillary HPLC.

### Protein identification by mass spectrometry

Protein identification, for 1D and/or 2D LC resolved peptides, was performed on reduced and alkylated, trypsin-digested *S. aureus* prepared as described above. HPLC recovered peptide pools were injected by direct infusion with a Nanomate (Advion BioSciences, Ithaca, NY), an automated chip-based nano-electrospray interface source coupled to a quadrupole–time of flight mass spectrometer, QStarXL MS/MS System (Applied Biosystems/Sciex, Framingham, MA). Computer-controlled data-dependent automated switching to MS/MS provided peptide sequence information. AnalystQS software (Applied Biosystems/Sciex) was used for data acquisition. Data processing and databank searching were performed with Mascot software (Matrix Science, Beachwood, OH) and a database containing forward and decoy sequences (RSUE database) that was constructed using the USA300_FPR3757 sequence.

***Identification of proteins*** **.** Bioinformatics analysis was carried out using the following online resources (reported in Table S1). Genome sequence; USA300_FPR3757 (NCBI accession #CP000255). Protein parameters (molecular mass, isoelectric point, GRAVY index); ProtParam tool, http://ca.expasy.org/tools/protparam.html [48]. Localization of proteins; PSORTb v.2.0, http://www.psort.org/ [11]. Membrane topology; HMMTOP, http://www.enzim.hu/hmmtop/ [49]. Signal sequence prediction; SignalP 3.0, http://www.cbs.dtu.dk/services/SignalP/ [50]; PrediSi, http://www.predisi.de/. Anchoring domain prediction; AUGUR, http://bioinfo.mikrobio.med.uni-giessen.de/augur/ [51]. Amino acid alignment; CLUSTALW2, http://www.ebi.ac.uk/Tools/clustalw2/index.html [52].

### Construction of isogenic *lukGH* deletion mutants (Δ*lukGH*)

Isogenic *lukGH* (*SAUSA300_1974* and *SAUSA300_1975* in USA300 strain FPR3757, NCBI accession #CP000255) deletion mutants (Δ*lukGH*) were generated in LAC wild-type and LACΔ*pvl* strains [23] using a previously described allelic replacement method [25]. Briefly, the 3′ region of the allelic replacement cassette was generated by PCR amplification of LAC genomic DNA with the primers 1973attB1-F and 1974*Sph*I-R, and the 5′ region with 1975*Sph*I-F and 1976attB2-R. PCR products were purified, digested with *Sph*I, ligated, and transferred into plasmid pKOR1 by recombination [25]. The resulting plasmid construct (pKOR1-Δ*lukGH*) was used for allelic replacement as described [25]. Primers used to construct Δ*lukGH* strains are as follows:

1973attB1-F: GGGGACAAGTTTGTACAAAAAAGCAGCATAAAAATATAGCAATAACTACATCCG

1974SphI-R:

GTTGCATGCTACATAGAATGTATGTAGG

1975SphI-F:

GTATCGGCATGCGAATAATATCACAAAAACAGAG

1976attB2-R:

GGGGACCACTTTGTACAAGAAAGTTGAACATAGGCGCAACATCTAATTCAT

Deletion of *lukGH* was confirmed in Δ*lukGH* and Δ*lukGH*/Δ*pvl* strains by PCR using primers 1973attB1-F and 1976attB2-R. PCR confirmation that Δ*pvl* was maintained during mutagenesis was carried out as previously described [23].

To generate the complemented gene deletion strains, DNA encoding the ribosome binding site and *lukGH* ORF were PCR-amplified from the USA300 genome and ligated into *BamH*I and *Nar*I sites of the *S. aureus* expression vector pTX-15 [53]. The resulting plasmid (p*lukGH*) was transformed into Δ*lukGH* and Δ*lukGH/*Δ*pvl* to produce Δ*lukGH*::p*lukGH* and Δ*lukGH/*Δ*pvl*::p*lukGH*. Primers used to construct the *lukGH* complementation vector are as follows:

pTX15_*lukGH*_F:

TTATTCACATGTCGAGGATCCTCAACAAATATCA

pTX15_*lukGH*_R:

ATTCTATGTAGGGCGCCACTTTTATTACTTATTTC

LukGH expression was confirmed by SDS-PAGE and immunoblot analysis as described below.

### SDS-PAGE and immunoblotting

LAC wild-type and isogenic Δ*pvl*, Δ*lukGH*, and Δ*lukGH*/Δ*pvl* deletion strains were cultured to early-stationary phase of growth in CCY media (OD_600_ = 2.0). Bacteria from 10 ml of culture were pelleted by centrifugation. Culture supernatant was aspirated, filter-sterilized, and stored at −80°C until use. Samples were boiled in Laemmli sample buffer [54] for 5 min and separated by 12.5% SDS-PAGE using Criterion precast polyacrylamide gels (Bio-Rad, Hercules, CA). Proteins were transferred to PVDF membranes using the iBlot transfer system (Invitrogen, Carlsbad, CA). Membranes were blocked using 10% goat serum in Tris-buffered saline containing 0.05% Tween-20 and antibody incubations were performed in blocking buffer diluted 1∶4 in TBS. Washes were performed in triplicate for 15 min each between antibody incubations using wash buffer (250 mM NaCl, 10 mM Hepes, and 0.2% Tween-20). LukG was detected using affinity-purified rabbit IgG directed against a peptide region of the protein (LWAKDNFTPKDKMP) (GenScript Corporation, Piscataway, NJ) and a donkey anti-rabbit IgG-HRP secondary antibody (Jackson ImmunoResearch, West Grove, PA). PVL was detected as described previously [26]. Proteins were visualized using a Supersignal West Pico Horseradish Peroxidase Detection Kit (Pierce Biotechnology, Rockford, IL) and X-ray film (Phenix Research Products, Chandler, NC).

### Subcellular localization of LukG

Bacteria from early-stationary phase (8 h) cultures were harvested by centrifugation (8,000× g for 10 min), washed with deionized water, and disrupted by high pressure (18,000 psi) using a French Press. The cell lysate was treated with Protease Inhibitor Cocktail and Nuclease Mix (Amersham Bioscience Corp, Piscataway, NJ) and the particulate fraction, which includes cell envelope components, was isolated by centrifugation (25,000× g for 30 min at 4°C). The clarified lysates, which contain cytoplasmic proteins, were stored at −80°C until used. The resulting pellet, containing cell envelope components, was resuspended in rehydration buffer (7 M urea, 2 M thiolurea, 2% triton X-100, 2 mM tributylphosphine, and 1% bromophenol blue) and incubated at ambient temperature for 2 h. These samples were clarified by centrifugation (25,000× g, 30 min) and proteins were analyzed by SDS-PAGE and immunoblotting (labeled as “membrane fraction” in Fig. 3A). Detergent-insoluble material (pellet from above) was incubated for 3 h in TE buffer (50 mM Tris-HCl and 10 mM EDTA, pH 8.0) with lysostaphin (40 µg/ml). These samples were clarified by centrifugation (25,000× g, 30 min) and soluble material was analyzed by SDS-PAGE (labeled as “lysostaphin extract” in Fig. 3A).

Culture supernatant proteins were precipitated with 9 vol of trichloroacetic acid:acetone (1∶8 v/v for 2 h at −20°C) and then analyzed by SDS-PAGE and immunoblotting as described above (labeled as “extracellular proteins” in Fig. 3A).

### Human PMN assays

Human neutrophils were isolated from heparinized venous blood of healthy donors using a published method [55]. PMN plasma membrane permeability (pore formation) was assessed by ethidium bromide (EtBr) uptake as described previously [23], [26]. Neutrophil lysis either after incubation with CCY culture supernatant or following phagocytosis of serum opsonized USA300 was measured by the release of lactate dehydrogenase (LDH) using a Cytotoxicity Detection Kit (Roche Applied Sciences, Pleasanton, California) as described previously [23], [26].

### Statistical analyses

All statistical analyses were performed using GraphPad Prism. Comparison of Mascot score distribution across subcellular components was performed using the Kruskal-Wallis test with Dunn's post-test. One-way or repeated-measures ANOVA followed by Tukey's post-test was used to assess significance in PMN pore formation and lysis assays.

## Supporting Information

Figure S1 USA300/LAC remains viable following trypsin digestion of surface proteins. (A) Bacteria were grown to the late-exponential phase of growth, harvested, washed, and resuspended in 0.6M sucrose and 2 mM DTT to promote exposure of surface proteins. Following addition of trypsin, colony-forming units were enumerated immediately (0 min) or after 60 min of incubation as described in methods. (B) Sypro Ruby-stained Tricine SDS-PAGE shows the low molecular weight tryptic peptides. The dominant bands at ∼15 kDa and ∼20 kDa represent singly autodigested trypsin and full-length trypsin, respectively.
(0.11 MB TIF)

Figure S2 Presence of LukG and LukH among S. aureus strains with published genomes and homology with other two-component leukotoxins. (A) Comparison of the DNA sequences of lukG and lukH with other sequenced S. aureus strains. (B) Inferred amino acid homology of LukG and LukH among sequenced S. aureus strains. (C) Homology of LukG and LukH with other S. aureus two-component leukotoxins. Alignments were generated by GeneDoc software v. 2.7. The nucleotide sequences for strains USA300_TCH1516, Newman, NCTC 8325, USA300_FPR3757, ED98, Mu3, JH1, JH9, N315, Mu50, RF122, MSSA476, MW2 and COL were obtained from NCBI database (GenBank accession no. CP000730, AP009351, CP000253, CP000255, CP001781, AP009324, CP000736, CP000703, BA000018, BA000017, AJ938182, BX571857, BA000033, CP000046, respectively). A gap, indicated by a bar, was introduced by the program CLUSTALW2 (www.ebi.ac.uk/Tools/clustalw2/index.html) to obtain maximal alignment of amino acids. Black shading indicates identical amino acids.
(0.25 MB DOC)

Table S1 USA300 proteins identified by surface proteomics. a) Cellular localization of protein as determined using PSORT v2.0. b) FPR3757 number corresponds to the gene number in the published FPR3757 genome [27]. c) Signal sequences were identified using the Gram-positive signal sequence prediction function on the SignalP 3.0 Server [28]. References 1. Movitz J (1974) A study on the biosynthesis of protein A in Staphylococcus aureus. Eur J Biochem 48: 131-136. 2. Foster SJ (1995) Molecular characterization and functional analysis of the major autolysin of Staphylococcus aureus 8325/4. J Bacteriol 177: 5723-5725. 3. Mazmanian SK, Skaar EP, Gaspar AH, Humayun M, Gornicki P, et al. (2003) Passage of heme-iron across the envelope of Staphylococcus aureus. Science 299: 906-909. 4. Zhang L, Jacobsson K, Strom K, Lindberg M, Frykberg L (1999) Staphylococcus aureus expresses a cell surface protein that binds both IgG and beta2-glycoprotein I. Microbiology 145 (Pt 1): 177-183. 5. Ni ED, Perkins S, Francois P, Vaudaux P, Hook M, et al. (1998) Clumping factor B (ClfB), a new surface-located fibrinogen-binding adhesin of Staphylococcus aureus. Mol Microbiol 30: 245-257. 6. Roche FM, Massey R, Peacock SJ, Day NP, Visai L, et al. (2003) Characterization of novel LPXTG-containing proteins of Staphylococcus aureus identified from genome sequences. Microbiology 149: 643-654. 7. Nugent KM, Huff E, Cole RM, Theodore TS (1974) Cellular location of degradative enzymes in Staphylococcus aureus. J Bacteriol 120: 1012-1016. 8. Jonsson K, Signas C, Muller HP, Lindberg M (1991) Two different genes encode fibronectin binding proteins in Staphylococcus aureus. The complete nucleotide sequence and characterization of the second gene. Eur J Biochem 202: 1041-1048. 9. Froman G, Switalski LM, Speziale P, Hook M (1987) Isolation and characterization of a fibronectin receptor from Staphylococcus aureus. J Biol Chem 262: 6564-6571. 10. Carneiro CR, Postol E, Nomizo R, Reis LF, Brentani RR (2004) Identification of enolase as a laminin-binding protein on the surface of Staphylococcus aureus. Microbes Infect 6: 604-608. 10.1016/j.micinf.2004.02.003 [doi];S1286457904000802 [pii]. 11. O'Brien L, Kerrigan SW, Kaw G, Hogan M, Penades J, et al. (2002) Multiple mechanisms for the activation of human platelet aggregation by Staphylococcus aureus: roles for the clumping factors ClfA and ClfB, the serine-aspartate repeat protein SdrE and protein A. Mol Microbiol 44: 1033-1044. 12. Kajimura J, Fujiwara T, Yamada S, Suzawa Y, Nishida T, et al. (2005) Identification and molecular characterization of an N-acetylmuramyl-L-alanine amidase Sle1 involved in cell separation of Staphylococcus aureus. Mol Microbiol 58: 1087-1101. MMI4881 [pii];10.1111/j.1365-2958.2005.04881.x [doi]. 13. McDevitt D, Francois P, Vaudaux P, Foster TJ (1994) Molecular characterization of the clumping factor (fibrinogen receptor) of Staphylococcus aureus. Mol Microbiol 11: 237-248. 14. Hussain M, Becker K, von Eiff C, Schrenzel J, Peters G, et al. (2001) Identification and characterization of a novel 38.5-kilodalton cell surface protein of Staphylococcus aureus with extended-spectrum binding activity for extracellular matrix and plasma proteins. J Bacteriol 183: 6778-6786. 15. Sakata N, Terakubo S, Mukai T (2005) Subcellular location of the soluble lytic transglycosylase homologue in Staphylococcus aureus. Curr Microbiol 50: 47-51. 10.1007/s00284-004-4381-9 [doi]. 16. Karlsson A, Saravia-Otten P, Tegmark K, Morfeldt E, Arvidson S (2001) Decreased amounts of cell wall-associated protein A and fibronectin-binding proteins in Staphylococcus aureus sarA mutants due to up-regulation of extracellular proteases. Infect Immun 69: 4742-4748. 10.1128/IAI.69.8.4742-4748.2001 [doi]. 17. Hammel M, Sfyroera G, Pyrpassopoulos S, Ricklin D, Ramyar KX, et al. (2007) Characterization of Ehp, a secreted complement inhibitory protein from Staphylococcus aureus. J Biol Chem 282: 30051-30061. M704247200 [pii];10.1074/jbc.M704247200 [doi]. 18. Yoshida A (1963) Staphylococcal delta-hemolysin. I. Purification and chemical properties. Biochim Biophys Acta 71: 544-553. 19. Wang R, Braughton KR, Kretschmer D, Bach TH, Queck SY, et al. (2007) Identification of novel cytolytic peptides as key virulence determinants for community-associated MRSA. Nat Med 13: 1510-1514. 20. Cuatrecasas P, Fuchs S, Anfinsen CB (1967) Catalytic properties and specificity of the extracellular nuclease of Staphylococcus aureus. J Biol Chem 242: 1541-1547. 21. Rooijakkers SH, Ruyken M, Roos A, Daha MR, Presanis JS, et al. (2005) Immune evasion by a staphylococcal complement inhibitor that acts on C3 convertases. Nat Immunol 6: 920-927. 22. Mollby R, Wadstrom T (1971) Separation of Gamma Hemolysin from Staphylococcus aureus Smith 5R. Infect Immun 3: 633-635. 23. Smeltzer MS, Gill SR, Iandolo JJ (1992) Localization of a chromosomal mutation affecting expression of extracellular lipase in Staphylococcus aureus. J Bacteriol 174: 4000-4006. 24. Stoll H, Dengjel J, Nerz C, Gotz F (2005) Staphylococcus aureus deficient in lipidation of prelipoproteins is attenuated in growth and immune activation. Infect Immun 73: 2411-2423. 73/4/2411 [pii];10.1128/IAI.73.4.2411-2423.2005 [doi]. 25. Downer R, Roche F, Park PW, Mecham RP, Foster TJ (2002) The elastin-binding protein of Staphylococcus aureus (EbpS) is expressed at the cell surface as an integral membrane protein and not as a cell wall-associated protein. J Biol Chem 277: 243-250. 26. Grundling A, Schneewind O (2007) Synthesis of glycerol phosphate lipoteichoic acid in Staphylococcus aureus. Proc Natl Acad Sci U S A 104: 8478-8483. 27. Diep BA, Gill SR, Chang RF, Phan TH, Chen JH, et al. (2006) Complete genome sequence of USA300, an epidemic clone of community-acquired meticillin-resistant Staphylococcus aureus. Lancet 367: 731-739. 28. Bendtsen JD, Nielsen H, von Heijne G, Brunak S (2004) Improved prediction of signal peptides: SignalP 3.0. J Mol Biol 340: 783-795. 10.1016/j.jmb.2004.05.028 [doi];S0022283604005972 [pii].
(0.20 MB DOC)

Table S2 The surface proteome (surfome) of USA300 strain LAC contains cell wall-associated, extracellular, membrane, and cytoplasmic proteins. A total of 113 proteins were identified in the LAC surfome. The peptide abundance ranking in Column A was determined by ranking the identified proteins by 1) Combined Mascot Score (Column G), then 2) Queries matched (Column E), then 3) Unique peptides matched (Column F). The FPR3757 number in Column C refers to the gene number in the SAUSA300_FPR3757 genome. The Percent Coverage (Column H) was determined by dividing the number of peptides identified by the total number of tryptic peptides in a given protein. SignalP 3.0 and PrediSi were used to determine if a Gram-positive signal sequence was present in each identified protein (Columns I and J). The GRAVY index (Column M) is a measure of the hydrophobicity of each identified protein. The PSORT localization (Column N) was assigned based upon the PSORT 2.0 localization for FPR3757. The experimental/knowledge localization (Column O) was assigned based upon 1) published data that demonstrate localization of the protein, or 2) PSORT 2.0 data if published data were not available. Whether a protein contained an anchoring domain was determined using AUGUR. The cell wall anchoring domain (Column P) was then identified by visual inspection of the primary protein sequence. The signal peptide cleavage site (Column Q) was identified using SignalP-NN.
(0.06 MB XLS)
