# Supplementary figures and images for: Identification of a Novel Staphylococcus aureus Two-Component Leukotoxin Using Cell Surface Proteomics
Source: PLoS One. 2010 Jul 16;5(7):e11634. doi: 10.1371/journal.pone.0011634 (PMC2905442; doi:10.1371/journal.pone.0011634)

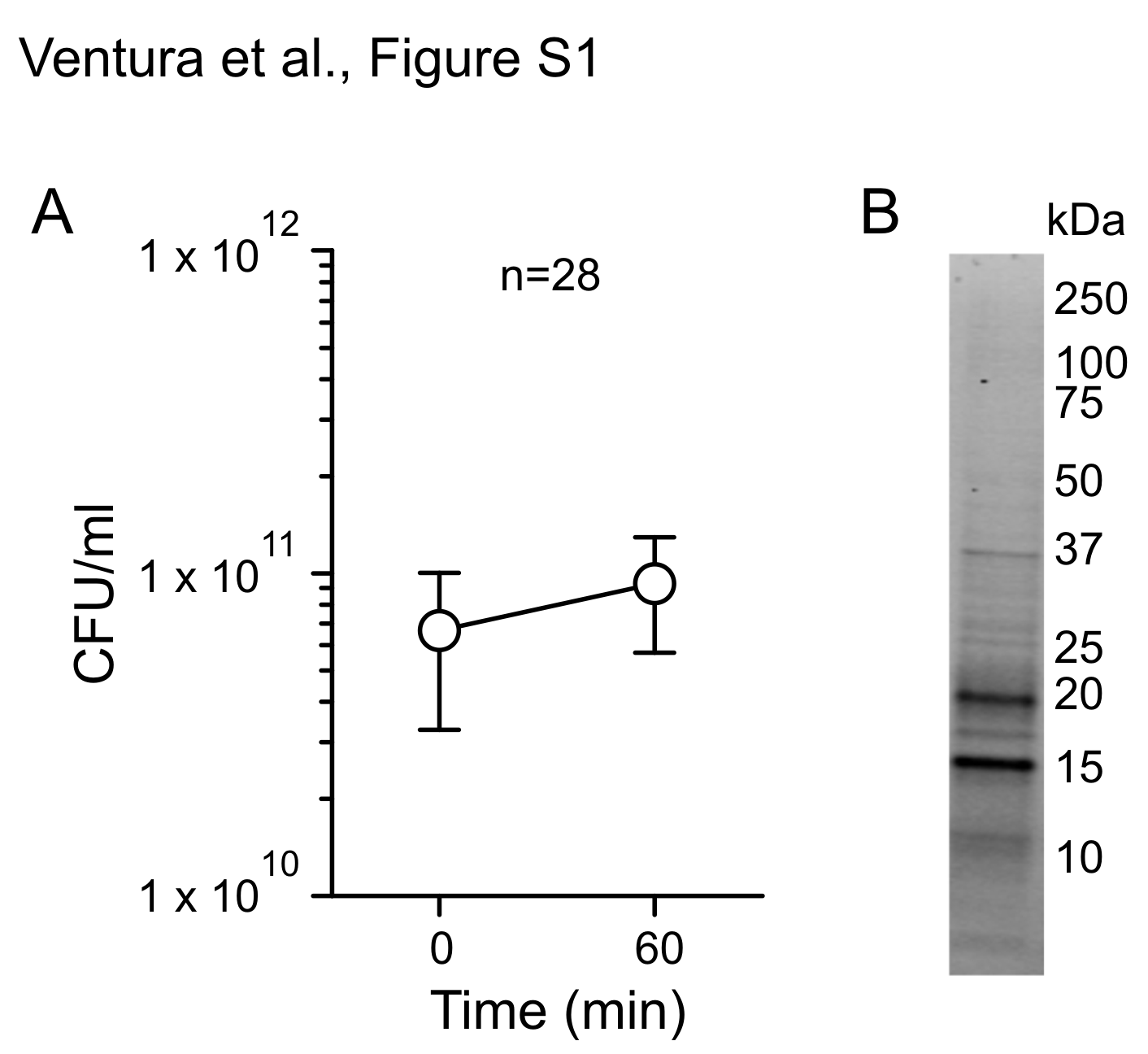

Supplement: Figure S1 — USA300/LAC remains viable following trypsin digestion of surface proteins. (A) Bacteria were grown to the late-exponential phase of growth, harvested, washed, and resuspended in 0.6M sucrose and 2 mM DTT to promote exposure of surface proteins. Following addition of trypsin, colony-forming units were enumerated immediately (0 min) or after 60 min of incubation as described in methods. (B) Sypro Ruby-stained Tricine SDS-PAGE shows the low molecular weight tryptic peptides. The dominant bands at ∼15 kDa and ∼20 kDa represent singly autodigested trypsin and full-length trypsin, respectively. (0.11 MB TIF) [file pone.0011634.s001.tif]
