# Supplementary material for: Identification of a Novel Staphylococcus aureus Two-Component Leukotoxin Using Cell Surface Proteomics
Source: PLoS One. 2010 Jul 16;5(7):e11634. doi: 10.1371/journal.pone.0011634 (PMC2905442; doi:10.1371/journal.pone.0011634)

Figure S2

1. Nucleotide alignment of *lukG* (*SAUSA300_1974*) and *lukH* (*SAUSA300_1975*) among *S. aureus* strains.

*lukG*
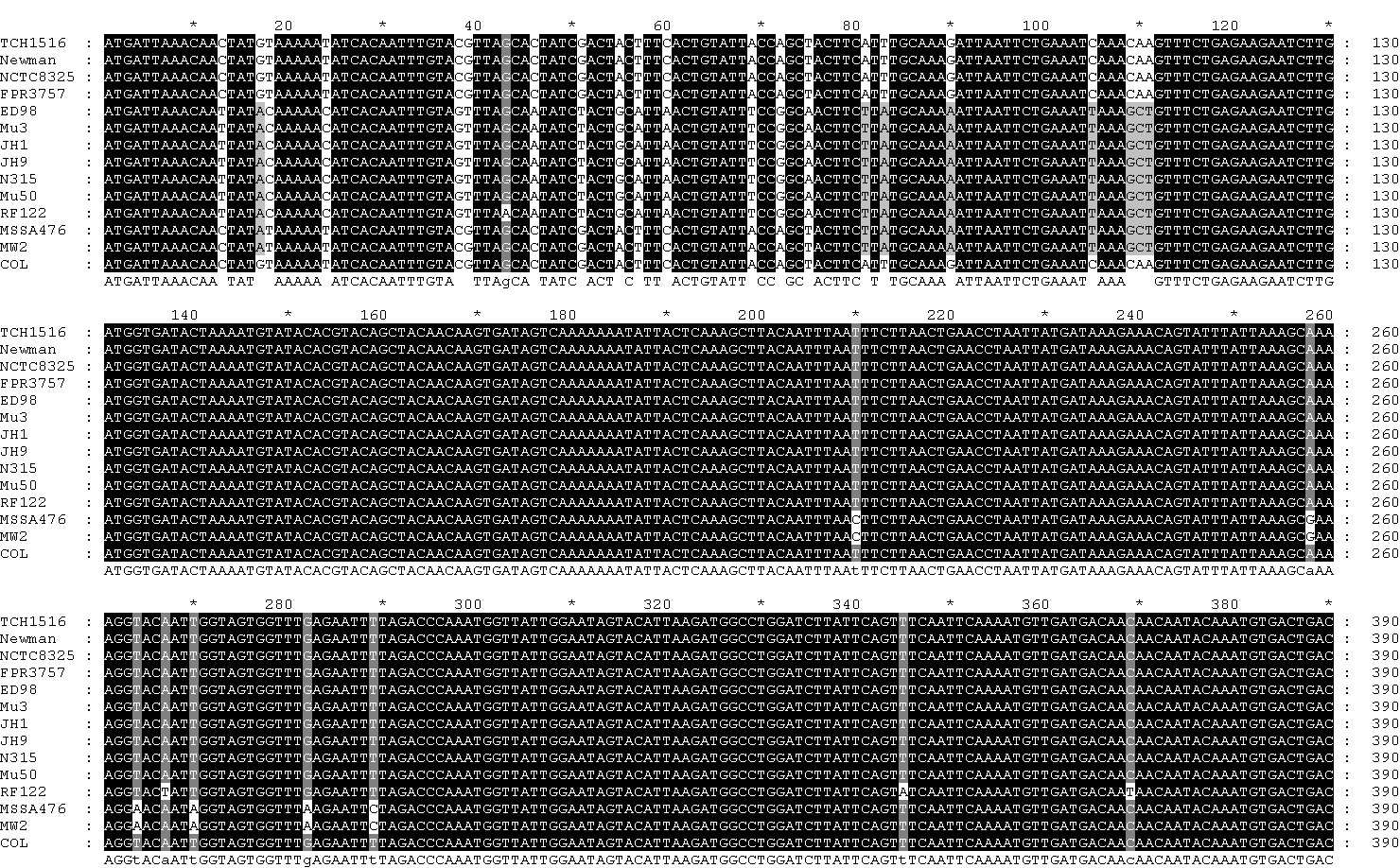


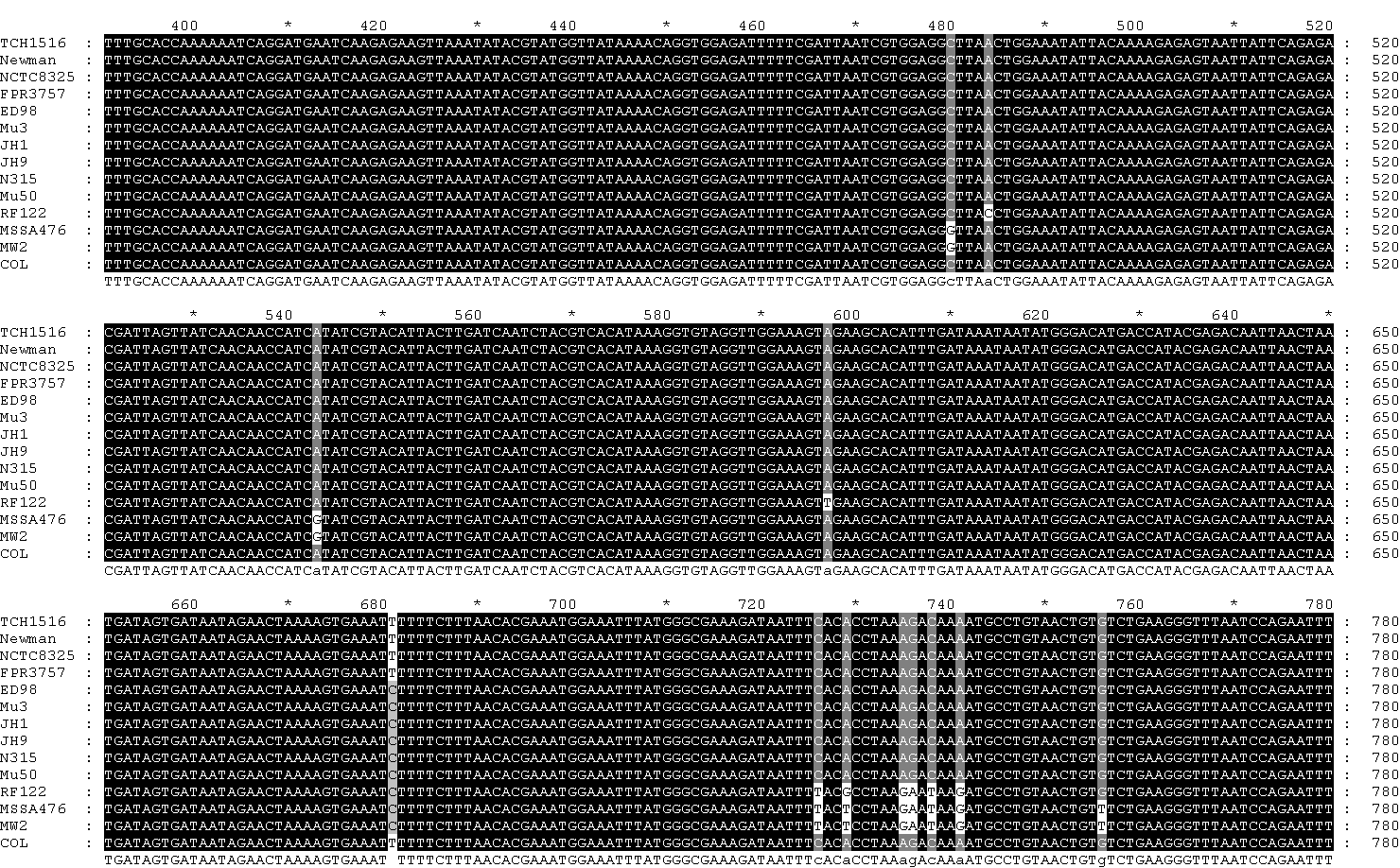


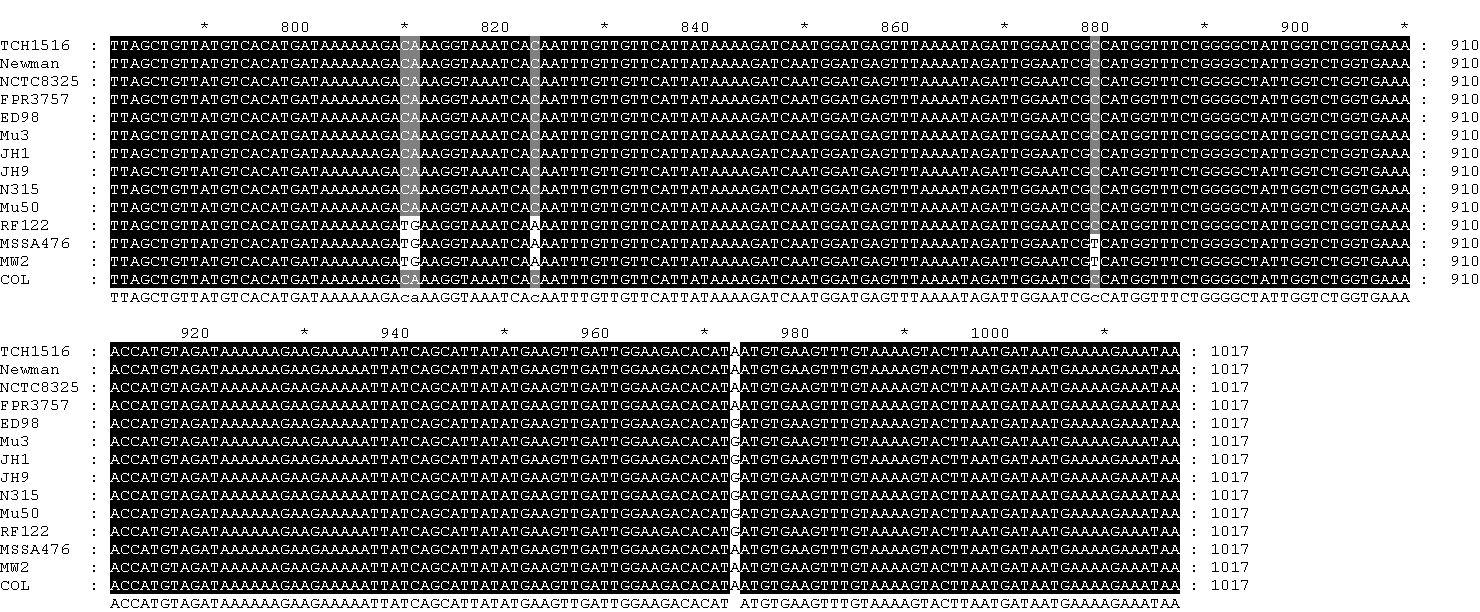


*lukH*
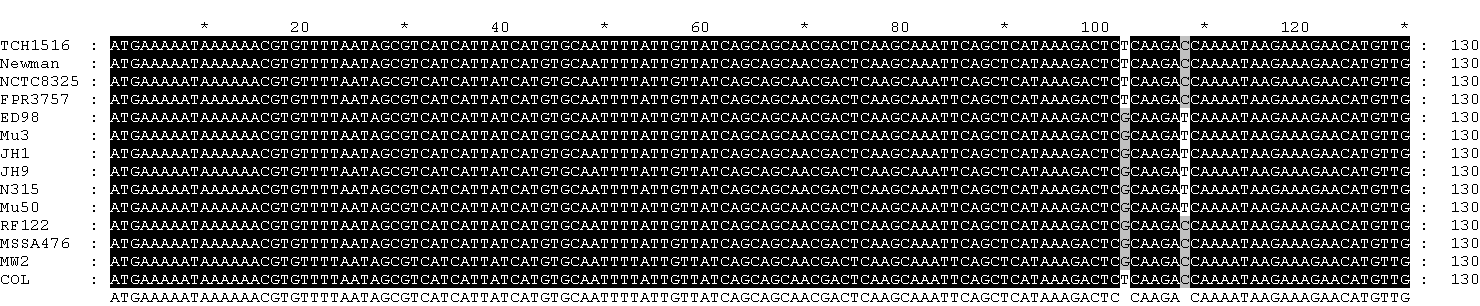


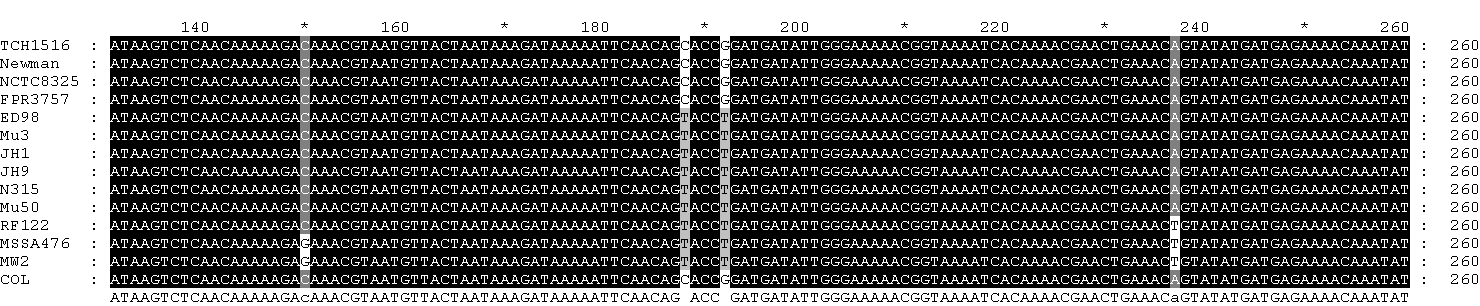


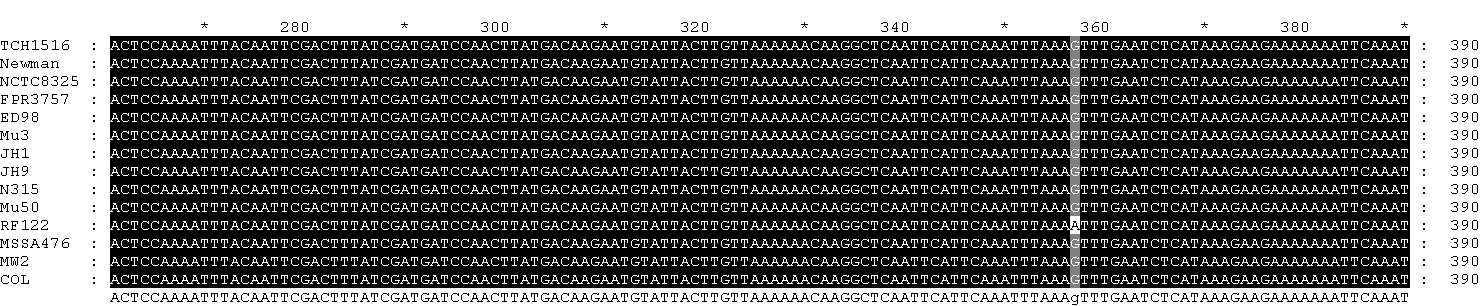


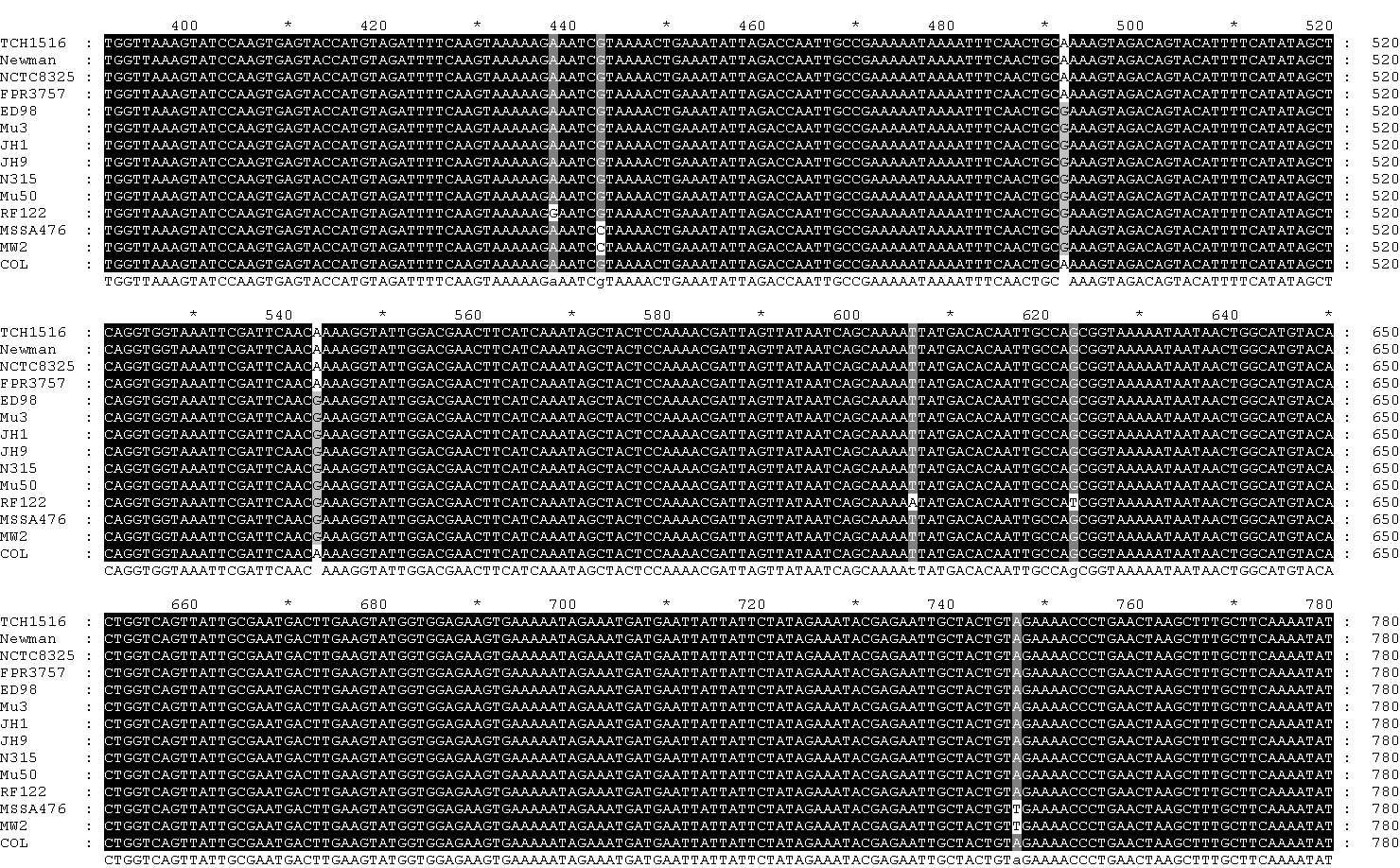


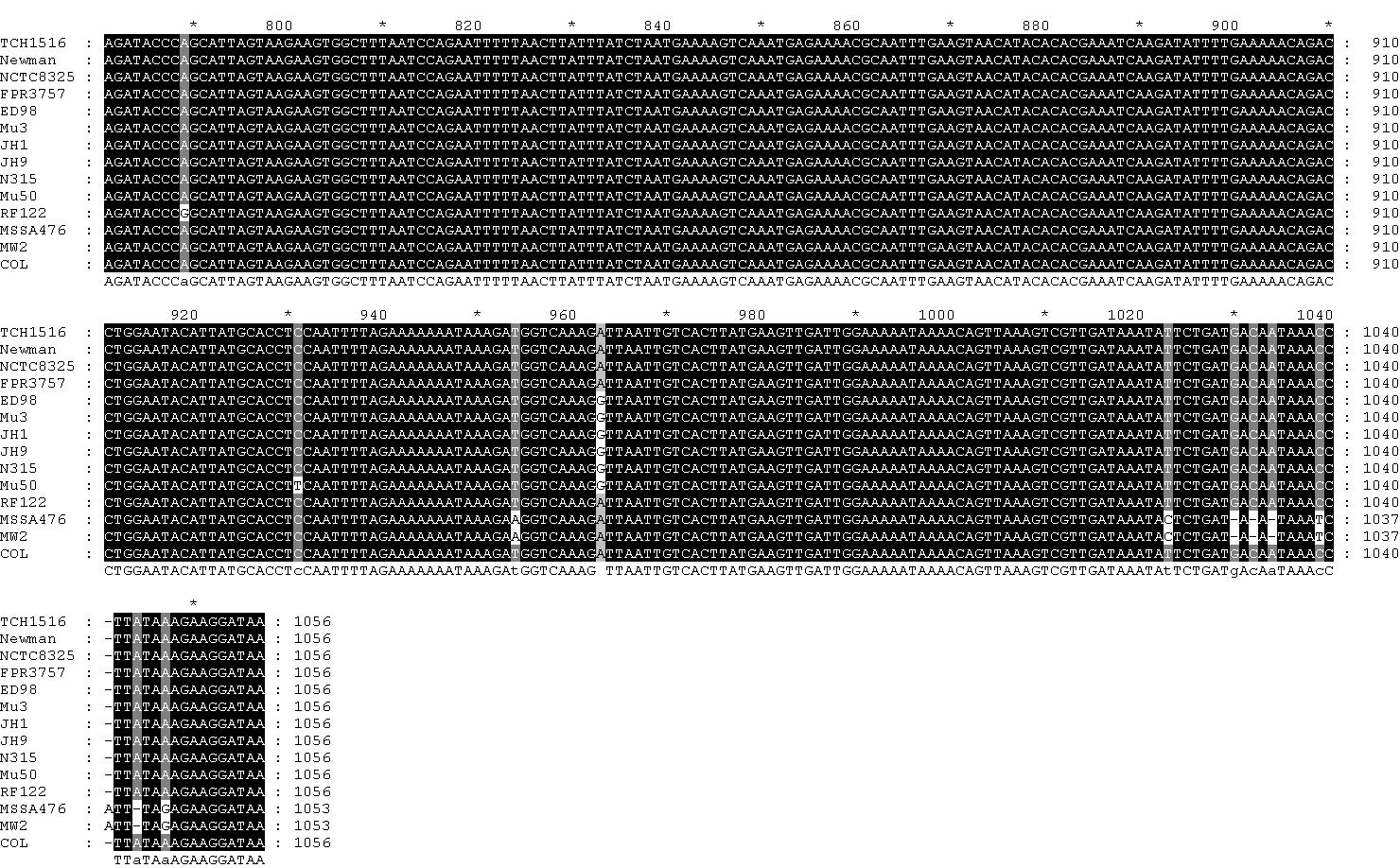


B. Amino acid alignment of LukGH among *S. aureus* strains.

LukG
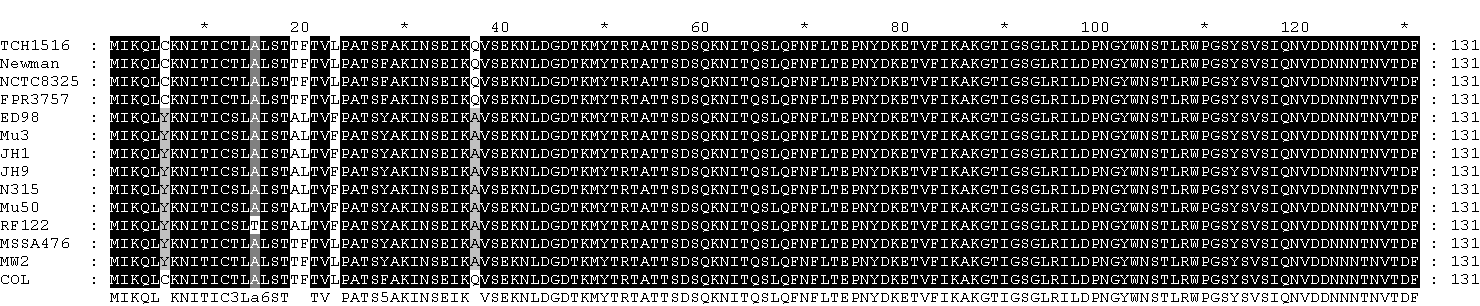


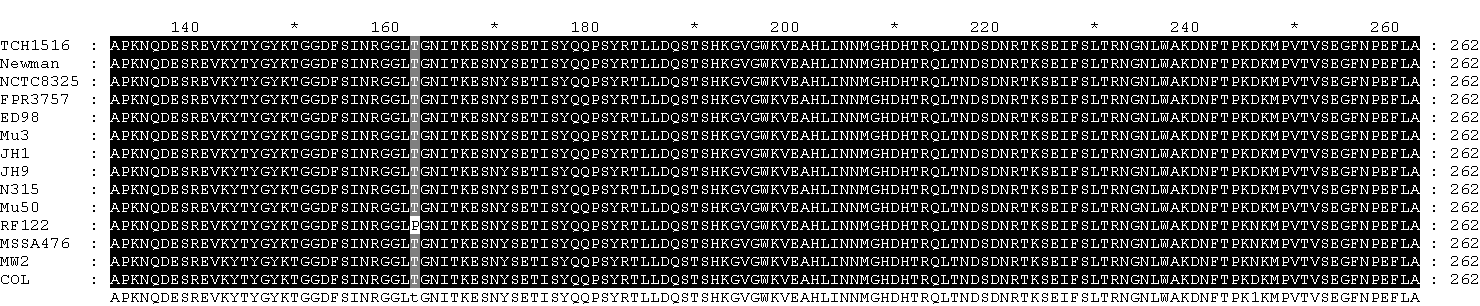


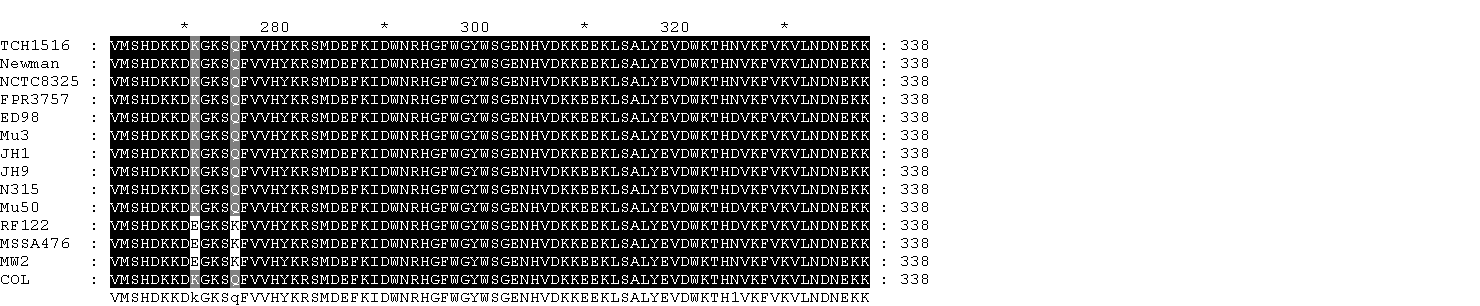


LukH
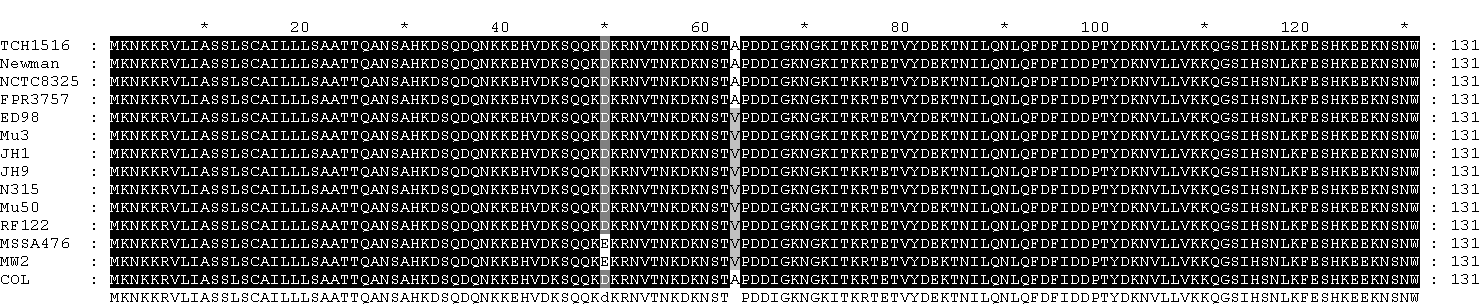


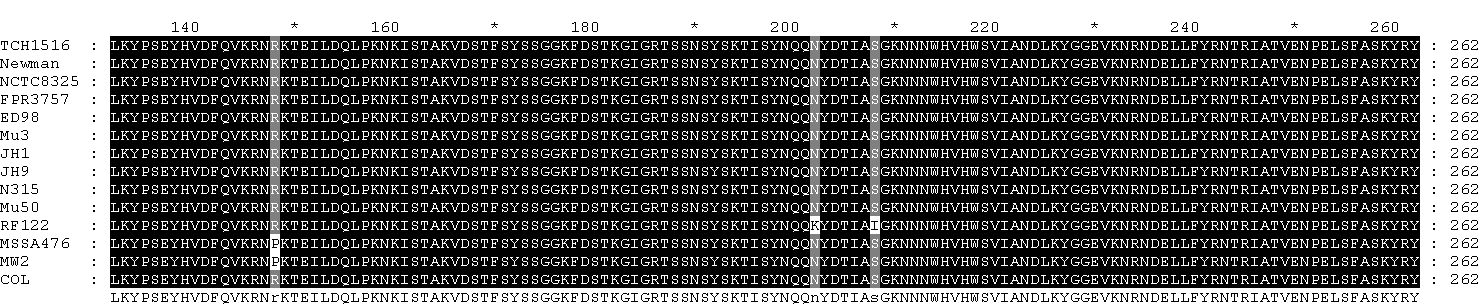


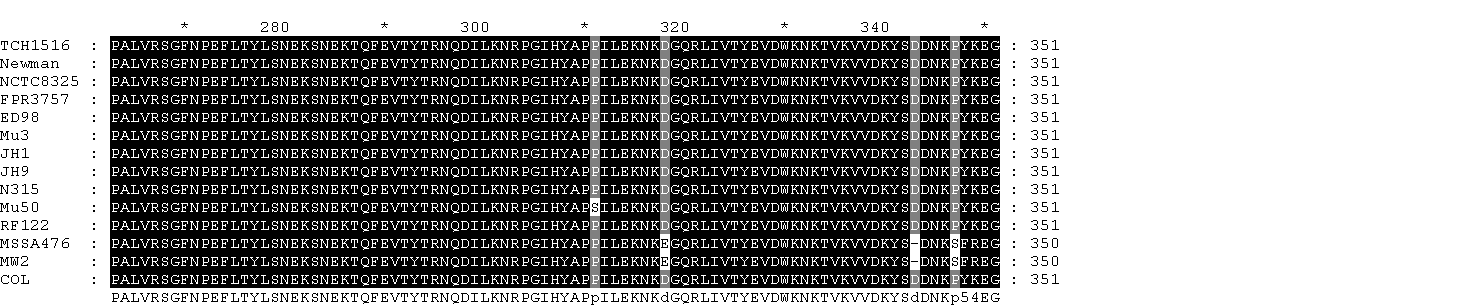


C. Amino acid homology of LukG and LukH with other USA300 two-component leukotoxins.


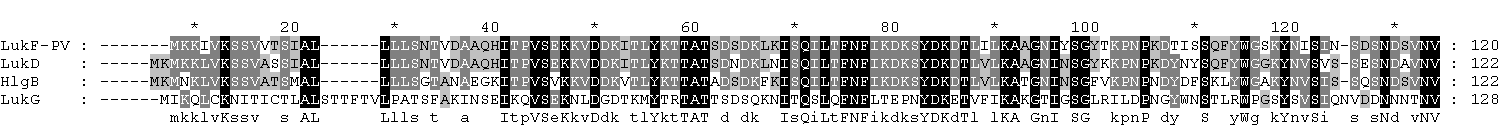


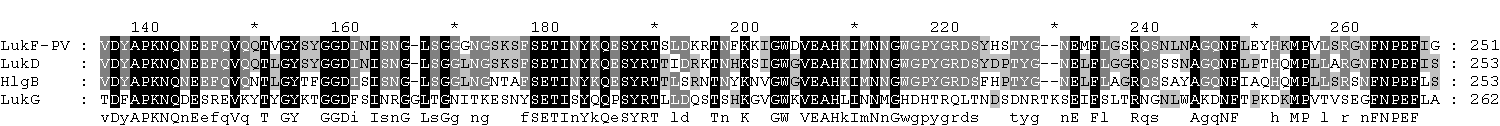


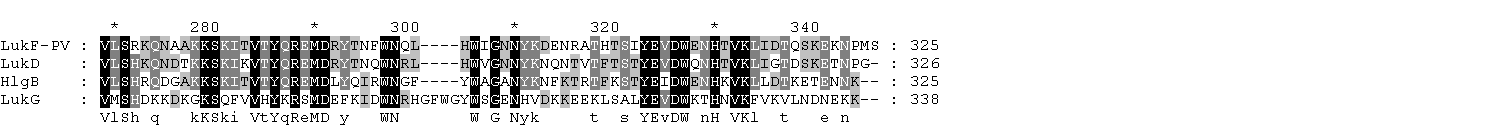


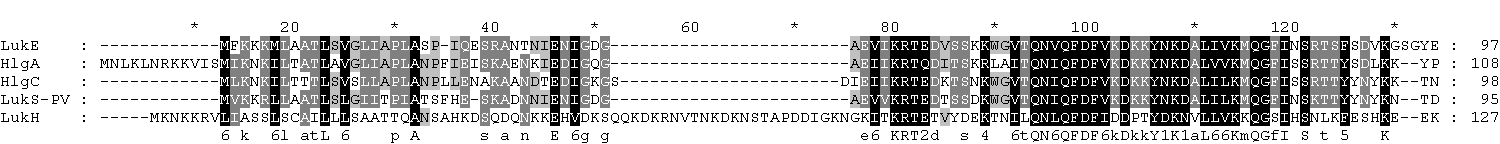


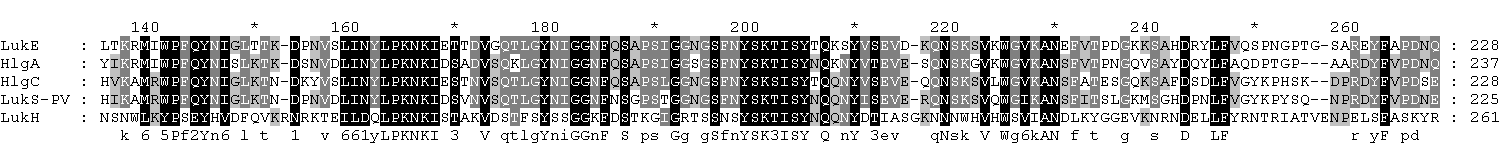


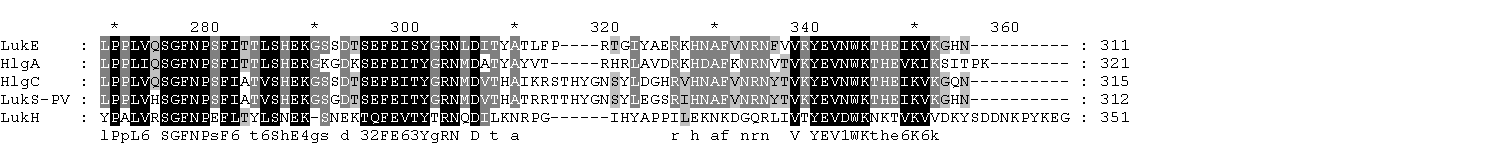

Supplement: Figure S2 — Presence of LukG and LukH among S. aureus strains with published genomes and homology with other two-component leukotoxins. (A) Comparison of the DNA sequences of lukG and lukH with other sequenced S. aureus strains. (B) Inferred amino acid homology of LukG and LukH among sequenced S. aureus strains. (C) Homology of LukG and LukH with other S. aureus two-component leukotoxins. Alignments were generated by GeneDoc software v. 2.7. The nucleotide sequences for strains USA300_TCH1516, Newman, NCTC 8325, USA300_FPR3757, ED98, Mu3, JH1, JH9, N315, Mu50, RF122, MSSA476, MW2 and COL were obtained from NCBI database (GenBank accession no. CP000730, AP009351, CP000253, CP000255, CP001781, AP009324, CP000736, CP000703, BA000018, BA000017, AJ938182, BX571857, BA000033, CP000046, respectively). A gap, indicated by a bar, was introduced by the program CLUSTALW2 (www.ebi.ac.uk/Tools/clustalw2/index.html) to obtain maximal alignment of amino acids. Black shading indicates identical amino acids. (0.25 MB DOC) [file pone.0011634.s002.doc]
