# Supplementary material for: Identification of a Novel Staphylococcus aureus Two-Component Leukotoxin Using Cell Surface Proteomics
Source: PLoS One. 2010 Jul 16;5(7):e11634. doi: 10.1371/journal.pone.0011634 (PMC2905442; doi:10.1371/journal.pone.0011634)
Supplement: Table S1 — USA300 proteins identified by surface proteomics. a) Cellular localization of protein as determined using PSORT v2.0. b) FPR3757 number corresponds to the gene number in the published FPR3757 genome [27]. c) Signal sequences were identified using the Gram-positive signal sequence prediction function on the SignalP 3.0 Server [28]. References 1. Movitz J (1974) A study on the biosynthesis of protein A in Staphylococcus aureus. Eur J Biochem 48: 131-136. 2. Foster SJ (1995) Molecular characterization and functional analysis of the major autolysin of Staphylococcus aureus 8325/4. J Bacteriol 177: 5723-5725. 3. Mazmanian SK, Skaar EP, Gaspar AH, Humayun M, Gornicki P, et al. (2003) Passage of heme-iron across the envelope of Staphylococcus aureus. Science 299: 906-909. 4. Zhang L, Jacobsson K, Strom K, Lindberg M, Frykberg L (1999) Staphylococcus aureus expresses a cell surface protein that binds both IgG and beta2-glycoprotein I. Microbiology 145 (Pt 1): 177-183. 5. Ni ED, Perkins S, Francois P, Vaudaux P, Hook M, et al. (1998) Clumping factor B (ClfB), a new surface-located fibrinogen-binding adhesin of Staphylococcus aureus. Mol Microbiol 30: 245-257. 6. Roche FM, Massey R, Peacock SJ, Day NP, Visai L, et al. (2003) Characterization of novel LPXTG-containing proteins of Staphylococcus aureus identified from genome sequences. Microbiology 149: 643-654. 7. Nugent KM, Huff E, Cole RM, Theodore TS (1974) Cellular location of degradative enzymes in Staphylococcus aureus. J Bacteriol 120: 1012-1016. 8. Jonsson K, Signas C, Muller HP, Lindberg M (1991) Two different genes encode fibronectin binding proteins in Staphylococcus aureus. The complete nucleotide sequence and characterization of the second gene. Eur J Biochem 202: 1041-1048. 9. Froman G, Switalski LM, Speziale P, Hook M (1987) Isolation and characterization of a fibronectin receptor from Staphylococcus aureus. J Biol Chem 262: 6564-6571. 10. Carneiro CR, Postol E, Nomizo R, Reis LF, Brentani RR (2004) Identificati [file pone.0011634.s003.doc]

**Table S1. USA300 proteins identified by surface proteomics.**

| **Localization^a)^** | **Protein** | **FPR3757 number^b)^** | **Mascot score** | **Signal sequence^c)^** | **Anchoring motif** | **Experimental localization** |
| --- | --- | --- | --- | --- | --- | --- |
| **Cell wall associated** | Immunoglobulin G binding protein A (Spa) | 0113 | 6910 | Yes | LPxTG, LysM | Cell wall [1] |
|  | Autolysin (Atl) | 0955 | 487 | Yes |  | Cell wall [2] |
|  | Iron-regulated surface determinant A (IsdA) | 1029 | 381 | Yes | LPxTG | Cell wall [3] |
|  | Immunoglobulin G binding protein Sbi | 2364 | 204 | Yes |  | Cell wall [4] |
|  | Clumping factor B (ClfB) | 2565 | 174 | Yes | LPxTG | Cell wall [5] |
|  | SasD | 0136 | 167 | Yes | LPxTG | Cell wall ][6] |
|  | 5’-nucleotidase | 0025 | 124 | Yes | LPxTG | Cell wall [7] |
|  | Iron-regulated surface determinant B (IsdB) | 1028 | 89 | Yes | LPxTG | Cell wall [3] |
|  | Fibronectin binding protein B (FnbB) | 2440 | 82 | Yes | LPxTG | Cell wall [8] |
|  | Fibronectin binding protein A (FnbA) | 2441 | 82 | Yes | LPxTG | Cell wall [9] |
|  | Enolase (Eno) | 0760 | 71 | No |  | Cell wall ][10] |
|  | Ser-Asp rich fibrinogen/bone sialoprotein-binding protein SdrE | 0548 | 58 | Yes | LPxTG | Cell wall [11] |
|  | N-acetylmuramyl-L-alanine amidase Sle1 | 0438 | 46 | Yes | LysM | Cell wall [12] |
|  | SasF | 2581 | 43 | Yes | LPxTG | Cell wall [6] |
|  | Clumping factor A (ClfA) | 0772 | 27 | Yes | LPxTG | Cell wall [13] |
|  | Iron-regulated surface determinant H (IsdH/HarA/SasI) | 1677 | 26 | Yes | LPxTG | Cell wall [6] |
|  | Extracellular matrix protein-binding protein (Emp) | 0774 | 22 | Yes |  | Cell wall [14] |
| **Extracellular** | **Leukotoxin S-H (LukS-H or LukH)** | **1975** | **1760** | **Yes** |  | **Extracellular (this study)** |
|  | Immunodominant staphylococcal antigen A (IsaA) | 2506 | 1044 | Yes |  | Cell wall, extracellular [15] |
|  | Staphylococcal secretory antigen A (SsaA) | 2249 | 599 | Yes |  |  |
|  | **Leukotoxin F-G (LukF-G or LukG)** | **1974** | **338** | **Yes** |  | **Extracellular (this study)** |
|  | Hypothetical exported protein | 0602 | 311 | Yes |  |  |
|  | Hypothetical exported protein | 0372 | 215 | Yes |  |  |
|  | Staphopain (ScpA) | 1890 | 208 | Yes |  | Extracellular [16] |
|  | Efb-homologous protein (Ehp) | 1052 | 157 | Yes |  | Extracellular [17] |
|  | Delta-hemolysin (Hld) | 1988 | 114 | No |  | Extracellular [18] |
|  | Phenol soluble modulin β1 | 1067 | 92 | No |  | Extracellular [19] |
|  | Thermonuclease (Nuc) | 0776 | 48 | Yes |  | Extracellular [20] |
|  | Staphylococcal complement inhibitor (SCIN) | 1919 | 47 | Yes |  | Extracellular [21] |
|  | N-acetylmuramoyl-L-alanine amidase | 2579 | 36 | Yes |  |  |
|  | Gamma-hemolysin component A (HlgA) | 2565 | 30 | Yes |  | Extracellular [22] |
|  | Hypothetical protein | 0992 | 28 | Yes |  |  |
|  | 5’-nucleotidase, acid phosphatase | 0307 | 23 | Yes |  | Extracellular [7] |
|  | Carboxy-terminal processing protease (CtpA) | 1313 | 22 | No |  |  |
|  | Lipase (Geh/Lip) | 2603 | 21 | Yes |  | Extracellular [23] |
|  | Triacylglycerol lipase | 0320 | 21 | Yes |  |  |
| **Membrane** | Peptidyl-prolyl cis-trans isomerase (PrsA) | 1790 | 135 | Yes |  | Membrane [24] |
|  | Elastin binding protein (EbpS) | 1370 | 100 | No | LysM | Membrane [25] |
|  | Hypothetical exported protein; putative lipoprotein | 0079 | 65 | Yes |  |  |
|  | MecA protein | 0032 | 46 | Yes |  |  |
|  | Hypothetical protein; membrane protein possibly part of ATP transporter | 0934 | 42 | Yes |  |  |
|  | ABC transporter permease protein | 2556 | 33 | Yes |  |  |
|  | Cell shape-determining protein MreC | 1605 | 29 | Yes |  |  |
|  | Glycerol phosphate lipoteichoic acid synthase (LtaS) | 0703 | 24 | Yes |  | Membrane [26] |
|  | Capsular polysaccharide biosynthesis chain length regulator (CapA) | 2598 | 22 | Yes |  |  |
|  | Manganese-binding protein PsaA/MntC/SitC | 0618 | 22 | Yes |  | Membrane [24] |
|  | Phosphonate ABC transporter substrate binding protein | 0145 | 22 | Yes |  |  |
|  | ABC transporter substrate binding protein | 0437 | 21 | Yes |  |  |
|  | LysE/YggA family protein; lysine efflux permease | 0784 | 20 | No |  |  |
|  | ATP binding protein (contains P loop) | 0748 | 19 | No |  |  |
| **Cytoplasmic** | Protein translation elongation factor Tu (EF-Tu, Tuf) | 0533 | 208 | No |  |  |
|  | Protein translation elongation factor G (EF-G, FusA) | 0532 | 77 | No |  |  |
|  | Triosephosphate isomerase (TpiA) | 0758 | 62 | No |  |  |
|  | Ribosomal protein L25P/general stress protein (Ctc) | 0479 | 57 | No |  |  |
|  | Ribosomal protein L10P | 0524 | 53 | No |  |  |
|  | Ribosomal protein S9P (RpsI) | 2171 | 52 | No |  |  |
|  | Malate:quinone oxidoreductase (Mqo2) | 2541 | 51 | No |  |  |
|  | Ribosomal protein L13P (RplM) | 2172 | 49 | No |  |  |
|  | Pyruvate dehydrogenase E1 component (PdhB) | 0994 | 48 | No |  |  |
|  | Ribosomal protein S2P (RpsB) | 1149 | 46 | No |  |  |
|  | Ribosomal protein S7P | 0531 | 44 | No |  |  |
|  | Ribosomal protein S3 (RpsC) | 2198 | 43 | No |  |  |
|  | Ribosomal protein S4P (RpsD) | 1666 | 42 | No |  |  |
|  | Ribosomal protein S12 (RpsL) | 0530 | 41 | No |  |  |
|  | Ribosomal protein L19P (RplS) | 1134 | 40 | No |  |  |
|  | ATP synthase (AtpA) | 2060 | 39 | No |  |  |
|  | Cell division protein FtsZ | 1080 | 38 | No |  |  |
|  | Glyceraldehyde-3-phosphate dehydrogenase (GapC) | 0756 | 38 | No |  |  |
|  | Ribosomal protein L6P (RplF) | 2189 | 37 | No |  |  |
|  | Ribosomal protein L22P (RplV) | 2199 | 34 | Yes |  |  |
|  | Methionyl-tRNA synthetase/protein secretion chaperonin (CsaA) | 0467 | 33 | No |  |  |
|  | Ribosomal protein L15 (RplO) | 2185 | 33 | No |  |  |
|  | Ribosomal protein S5P (RpsE) | 2187 | 32 | No |  |  |
|  | Ribosomal protein L24P (RlpX) | 2193 | 32 | No |  |  |
|  | Ribosomal protein L12P (L7/L12) | 0525 | 29 | No |  |  |
|  | Transcriptional regulator, TetR family (AcrR) | 2322 | 29 | No |  |  |
|  | DNA-binding protein HU (Hup) | 1362 | 29 | No |  |  |
|  | DNA polymerase III, delta subunit (HolA) | 1546 | 27 | No |  |  |
|  | Enoyl-[acyl-carrier protein] reductase (FabI) | 0912 | 27 | No |  |  |
|  | Ribosomal protein S13P (RpSM) | 2180 | 27 | No |  |  |
|  | Protein translation elongation factor Ts (EF-Ts, Tsf) | 1150 | 26 | No |  |  |
|  | Inosine-5’-monophosphate dehydrogenase | 0388 | 25 | No |  |  |
|  | Ribosomal protein L17P (RplQ) | 2177 | 24 | No |  |  |
|  | Uracil phosphoribosyltransferase (Upp) | 2066 | 24 | No |  |  |
|  | Alkaline shock protein (Asp23) | 2142 | 23 | No |  |  |
|  | Hypothetical/putative pit accessory protein | 0649 | 23 | No |  |  |
|  | Mevalonate kinase (MvaK1) | 0572 | 23 | No |  |  |
|  | Poly(glycerol-phosphate) alpha-glucosyltransferase | 0939 | 23 | No |  |  |
|  | Sigma factor sigB regulation protein RsbU | 2025 | 23 | No |  |  |
|  | Transcriptional regulator, LytR family | 0958 | 23 | Yes |  |  |
|  | Biotin operon repressor/Biotin-(acetyl-CoA carboxylase) ligase (BirA) | 1347 | 22 | No |  |  |
|  | Conserved hypothetical protein | 0192 | 22 | No |  |  |
|  | Ferric anguibactin transporter ATP binding protein (SstC) | 0720 | 22 | No |  |  |
|  | Fructose-bisphosphate aldolase (FbaA) | 2079 | 22 | No |  |  |
|  | Hypothetical protein | 1744 | 22 | No |  |  |
|  | Acetyl-CoA acetyltransferase | 0355 | 21 | No |  |  |
|  | Competence/damage-inducible protein CinA | 1177 | 21 | No |  |  |
|  | Hypothetical protein | 1750 | 21 | No |  |  |
|  | Replicative DNA helicase | 0016 | 21 | No |  |  |
|  | Ribosomal protein L23 (RplW) | 2202 | 21 | No |  |  |
|  | Ribosomal protein S10P (RpsJ) | 2205 | 21 | Yes |  |  |
|  | Ribosomal protein S16P (RpsP) | 1131 | 21 | Yes |  |  |
|  | DNA helicase II (PcrA) | 1886 | 20 | No |  |  |
|  | Glycerol-3-phosphate cytidylyltransferase (TagD) | 0628 | 20 | No |  |  |
|  | Proline dipeptidase | 1654 | 20 | No |  |  |
|  | Pyrodoxal biosynthesis lyase PdxS | 0504 | 20 | No |  |  |
|  | Ribosomal protein L3P (RplC) | 2204 | 20 | No |  |  |
|  | RNA-metabolising metallo-beta-lactamase | 1168 | 20 | No |  |  |
|  | DNA polymerase I (PolA) | 1636 | 19 | No |  |  |
|  | Hydrolase, haloacid dehalogenase-like, subfamily IA | 0557 | 19 | No |  |  |
|  | Transcriptional regulator, LysR family | 0093 | 19 | No |  |  |
|  | Hypothetical cytosolic protein | 0198 | 18 | No |  |  |
|  | Putative helix-turn-helix DNA binding protein | 1129 | 18 | No |  |  |
